# TOR functions as a molecular switch connecting an iron cue with host innate defense against bacterial infection

**DOI:** 10.1371/journal.pgen.1009383

**Published:** 2021-03-03

**Authors:** Yi-Cheng Ma, Li-Li Dai, Bei-Bei Qiu, Ying Zhou, Yu-Qiang Zhao, Yu Ran, Ke-Qin Zhang, Cheng-Gang Zou

**Affiliations:** 1 State Laboratory for Conservation and Utilization of Bio-Resources in Yunnan, School of Life Sciences, Yunnan University, Kunming, Yunnan, China; 2 School of Agronomy and Life Sciences, Kunming University, Kunming, Yunnan, China; 3 College of Chemical Science and Technology, Yunnan University, Kunming, Yunnan, China; University of Massachusetts Medical School, UNITED STATES

## Abstract

As both host and pathogen require iron for survival, iron is an important regulator of host-pathogen interactions. However, the molecular mechanism by which how the availability of iron modulates host innate immunity against bacterial infections remains largely unknown. Using the metazoan *Caenorhabditis elegans* as a model, we demonstrate that infection with a pathogenic bacterium *Salmonella enterica* serovar Typhimurium induces autophagy by inactivating the target of rapamycin (TOR). Although the transcripts of *ftn-1* and *ftn-2* encoding two H-ferritin subunits are upregulated upon *S*. Typhimurium infection, the ferritin protein is kept at a low level due to its degradation mediated by autophagy. Autophagy, but not ferritin, is required for defense against *S*. Typhimurium infection under normal circumstances. Increased abundance of iron suppresses autophagy by activating TOR, leading to an increase in the ferritin protein level. Iron sequestration, but not autophagy, becomes pivotal to protect the host from *S*. Typhimurium infection in the presence of exogenous iron. Our results show that TOR acts as a regulator linking iron availability with host defense against bacterial infection.

## Author summary

Iron, an essential nutrient for both hosts and pathogens, can impact host-pathogen interactions. Thus, depending on availability of iron, hosts may use distinct strategies to defend against bacterial infections. Using the model organism *Caenorhabditis elegans*, we show that autophagy is activated and required for resistance to *S*. Typhimurium infection under normal conditions. In the presence of exogenous iron, autophagy is suppressed, leading to an increase in the protein level of ferritin, which in turn protects worms against *S*. Typhimurium infection. Finally, our data demonstrate that TOR is a molecular switch for turning autophagy and ferritin on and off. Thus, our study reveals a previously unknown role of TOR in modulating the transition between autophagy and iron sequestration in response to a bacterial infection via sensing an iron cue.

## Introduction

Iron is widely involved in a variety of fundamental physiological processes, including DNA synthesis, mitochondrial respiration, oxygen transport, and heme synthesis. Iron homeostasis is tightly regulated to prevent cellular damage associated with iron overload or iron deficiency by a complex mechanism [1]. After entering the cytoplasm, iron can be stored in the iron-storage protein ferritin, or be exported to the extracellular fluid through the basolateral membrane transporter ferroportin [2,3]. After sequestering large amounts of Fe(II), heavy chain (H) ferritin oxidizes Fe(II) to Fe(III) by its ferroxidase activity [4]. In mammals, iron regulatory proteins 1 and 2 (IRP1 and IRP2) sense iron levels to regulate expression of iron metabolism-related genes at the post-transcriptional levels [5,6]. In the absence of iron, IRPs bind to iron-responsive elements in the promoter in the mRNAs of iron metabolism-related genes, such as ferritin and transferrin receptor, thus increasing iron uptake and decreasing iron sequestration. The target of rapamycin (TOR), a conserved Ser/Thr kinase, is an important sensor of various nutrients, such as amino acids and glucose [7]. As a major hub for integration of multiple environmental and metabolic signals, TOR regulates cell and organismal physiology by activating an array of anabolic processes such as protein synthesis and ribosome biogenesis, and by inhibiting catabolic processes such as autophagy in a diversity of eukaryotes [8]. Accumulating evidence demonstrates that the TOR signaling is also a regulator of iron homeostasis by modulating iron transporter and cellular iron flux [9].

As an important iron-storage protein, ferritin expression is regulated by iron by transcriptional and post-transcriptional mechanisms [10–12]. In vertebrates, ferritin induction by iron is mainly regulated at the translational level. When iron is depleted, IRP1 and IRP2 bind to the iron responsive elements in ferritin mRNA, thus inhibiting ferritin translation. The invertebrate organism *Caenorhabditis elegans* expresses two ferritins, *ftn-1* and *ftn-2*, which are orthologous to ferritin-H subunits [11,12]. The expression of *ftn-1* and *ftn-2* is regulated by iron at the transcriptional level: iron supplementation upregulates *ftn-1* and *ftn-2* expression, while iron chelation has the opposite effect [11]. Besides iron, pathogenic bacteria can also influence ferritin expression. It has been reported that the Gram-positive pathogen *Staphylococcus aureus* infection downregulates the mRNA levels of *ftn-1* in *C*. *elegans* [13]. In contrast, using a quantitative proteomics approach, Simonsen et al. identified that the protein level of FTN-2 was increased in *C*. *elegans* upon *Escherichia coli* strain LF82 isolated from patients with Crohn’s disease [14]. Importantly, *ftn-2* is required for resistance to infection with *E*. *coli* LF82 or *S*. *aureus* in worms.

Iron can also impact host immunity [15]. On the one hand, iron is required for generating the NADPH-dependent oxidative burst with reactive oxygen to kill bacterial pathogens by phagocytic cells [16]. On the other hand, oral iron supplementation increases the risk of bacterial infections by disrupting the gut epithelial integrity in humans and mice [17–19]. Additionally, iron overload reduces production of inflammatory factors, such as tumor necrosis factor-α and interleukin-6, in macrophages after treatment with lipopolysaccharide or *S*. Typhimurium infection [20,21]. After challenged with the pathogenic bacterium *Citrobacter rodentium*, mice fed iron-deficient diet have a lower grade of colon pathology, compared with those fed a high-iron diet [22]. Furthermore, the survival rate of *C*. *elegans* infected with *S*. Typhimurium is reduced by iron supplementation [22]. These data suggest that exogenous iron deteriorates host pathology upon bacterial infections. However, how iron availability regulates host innate immune responses to bacterial infections is not fully understood.

Using *C*. *elegans* as a model, we showed here that TOR was a key node in regulating the transition between autophagy and iron sequestration during infection by *S*. Typhimurium. Under normal conditions, *S*. Typhimurium infection induced autophagy by inhibiting TOR activity. Autophagy was required for resistance to *S*. Typhimurium infection. In the presence of exogenous iron, autophagy was suppressed due to activation of TOR. Inhibition of autophagy led to an increase in the protein level of ferritin, which in turn protect worms against *S*. Typhimurium infection.

## Results

### Exogenous iron accelerates worm death by inhibiting autophagy during S. Typhimurium infection

**Fig 1:**
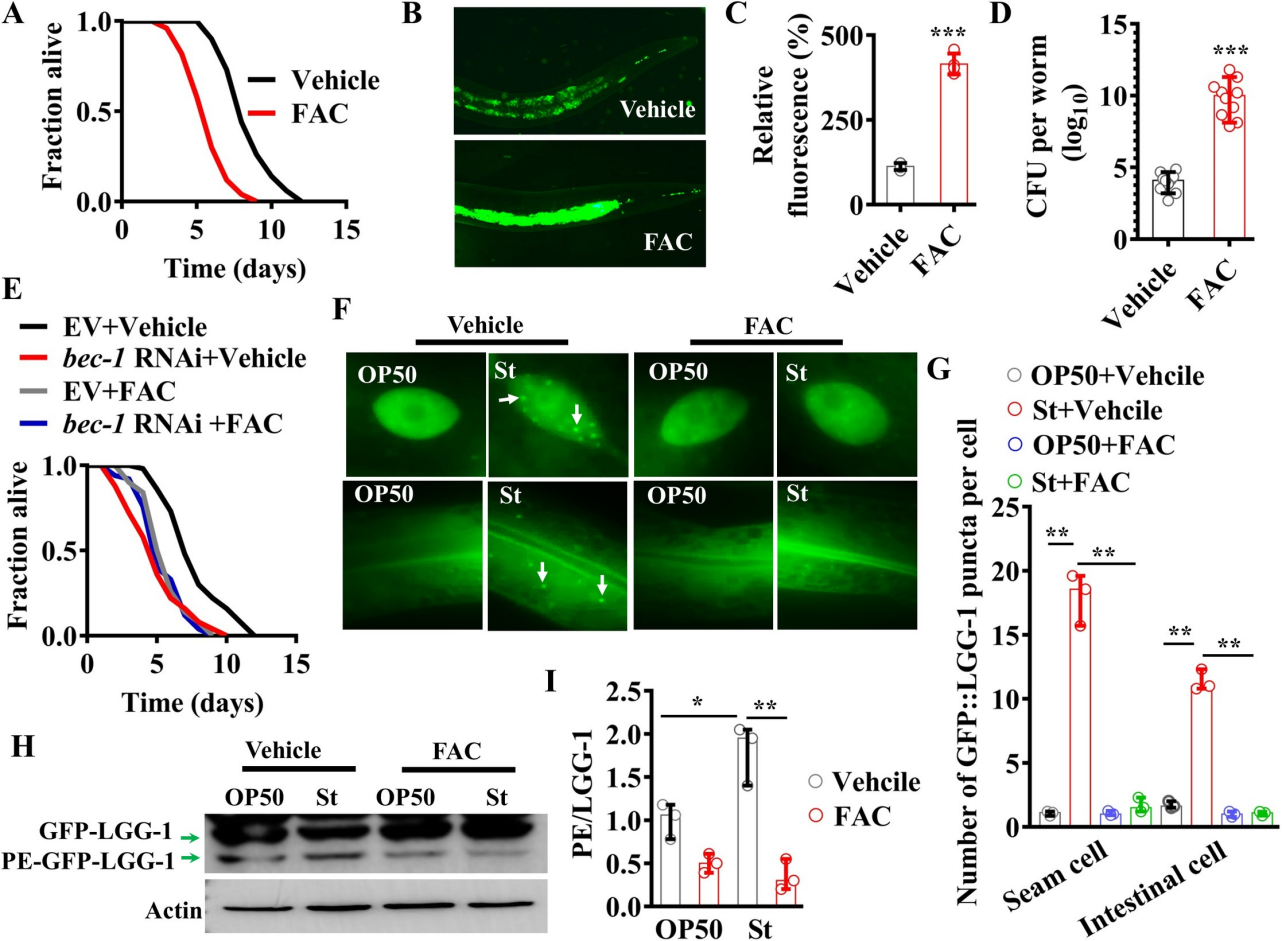
Iron inhibits autophagy induced by *S*. Typhimurium. **(A)** Wild-type (WT) worms treated with ferric ammonium citrate (FAC, 100 μM) were more susceptible to killing by *S*. Typhimurium (St) than those without FAC treatment (vehicle). *P*< 0.01 relative to vehicle (Log-rank test). Underlying data are available in S1 Table. **(B)** FAC treatment promoted accumulation of *S*. Typhimurium. Representative images of WT worms exposed to *S*. Typhimurium expressing GFP for 48 h. **(C)** Fluorescence intensity of *S*. Typhimurium expressing GFP. These results are mean ± SD of four independent experiments performed in triplicate (n = 15 worms per experiment). ***P< 0.001 (two sample t-test). Underlying data are available in S2 Table. **(D)** Colony-forming units (CFU) of *S*. Typhimurium were measured in worms. These results are mean ± SD of 10 independent experiments (n ≥ 50 worms per experiment). ***P< 0.001 (two sample t-test). Underlying data are available in S2 Table. **(E)** The survival of worms subjected to *bec-1* RNAi or empty vector (EV) after *S*. Typhimurium infection in the absence or presence of FAC. *P*< 0.01 relative to EV+ vehicle. Underlying data are available in S1 Table. **(F)** Representative images of autophagosomes (GFP::LGG-1 puncta) in the seam cells (upper panels) and intestinal cells (lower panels) of worms exposed to *S*. Typhimurium or *E*. *coli* OP50 for 12 h in the absence or presence of FAC. The arrows denote representative autophagosomes. **(G)** The numbers of GFP::LGG-1 puncta were counted. These results are mean ± SD of three independent experiments (n = 15 worms per experiment). **P< 0.01 (one-way ANOVA followed by a Student-Newman-Keuls test). Underlying data are available in S2 Table. **(H)** The protein levels of PE-GFP-LGG-1 to GFP-LGG-1 were measured by Western blot in worms exposed to *S*. Typhimurium or *E*. *coli* OP50 for 12 h in the absence or presence of FAC. The blot shown here is typical of three independent experiments. **(I)** Quantification of the ratio of PE-GFP-LGG-1 to GFP-LGG-1 from Western blot (H). These results are mean ± SD of three experiments. *P < 0.05, St versus *E*. *coli* OP50; **P < 0.01, St+FAC versus St alone (one-way ANOVA followed by a Student-Newman-Keuls test). Underlying data are available in S2 Table.

We first investigated the effect of iron on the pathogenicity of *S*. Typhimurium in *C*. *elegans*. Consistent with a previous observation [22], supplementation with ferric ammonium citrate (FAC, 100 μM) significantly reduced the survival rate of wild-type (WT) worms (Fig 1A). To determine whether the susceptibility is attributable to accelerated bacterial infection in the presence of exogenous iron, we infected worms with *S*. Typhimurium expressing GFP. We found that the accumulation of *S*. Typhimurium expressing GFP in the intestine of WT worms in the presence of exogenous iron was markedly higher than that under normal conditions (Fig 1B and 1C). Furthermore, iron supplementation markedly increased the colony forming units (CFU) of *S*. Typhimurium in WT worms (Fig 1D). Thus, the increased mortality of worms by exogenous iron is associated with an increase in *S*. Typhimurium colonization in worms. Meanwhile, we found that pharyngeal pumping and defecation rates in WT worms exposed to *S*. Typhimurium were comparable to those in worms fed standard food (the bacterium *E*. *coli* strain OP50) in the presence of 100 μM FAC (S1A and S1B Fig). Thus, increased *S*. typhimurium load in the presence of exogenous iron is not due to decreased defecation or increased feeding.

Autophagy plays an important role in host defense against bacterial infections in worms [23–27]. For instance, Jia et al. have reported that autophagy is involved in defense against *S*. Typhimurium infection in worms [23,26]. Consistent with this observation, knockdown of *bec-1*, an autophagic gene, by RNAi reduced the survival of WT worms after *S*. Typhimurium infection (Fig 1E). Interestingly, knockdown of *bec-1* by RNAi did not further increase the mortality of worms in the presence of 100 μM FAC (Fig 1E). One possibility is that the autophagic activity is inhibited in the presence of exogenous iron. To test this hypothesis, we analyzed autophagic activity by using transgenic worms carrying GFP::LGG-1 as the appearance of GFP::LGG-1-containing puncta is an indicator of autophagy in worms [28]. PE-LGG-1 accumulates on autophagic structures and contributes to the formation of punctate structures [28]. We found that the abundance of GFP::LGG-1-containing puncta was significantly increased in the seam and intestinal cells of worms after *S*. Typhimurium infection (Fig 1F and 1G). Iron supplementation suppressed this increase in GFP::LGG-1-containing puncta in the seam and intestinal cells (Fig 1F and 1G). Meanwhile, we also detected autophagy by measuring the ratio of PE-GFP-LGG-1 to GFP-LGG-1 by Western blot. A significant increase in the ratio of PE-GFP-LGG-1 to GFP-LGG-1 was observed in WT worms after *S*. Typhimurium infection (Fig 1H and 1I). Likewise, this increase was inhibited by iron supplementation (Fig 1F–1I). The increase in GFP::LGG-1-containing puncta by *S*. Typhimurium infection could result from either an induction of autophagy or a block in the turnover of LGG-1-bound autophagosomes. To distinguish between these possibilities, worms were injected with bafilomycin A1 (BafA), an inhibitor of lysosomal acidification, according to the method published by Wilkinson et al [29]. We found that injection of worms with BafA led to a robust increase in the number of GFP::LGG-1 puncta in worms after *S*. Typhimurium infection (S2A Fig), indicating induction of autophagic flux. SQST-1, the *C*. *elegans* orthologue for SQSTM1/p62 and an autophagy adaptor protein, is degraded upon induction of autophagy [28,30]. We thus determined the protein levels of p62/SQST-1 in transgenic worms carrying SQST-1::GFP by Western blot. We found that the protein levels of SQST-1::GFP were reduced after *S*. Typhimurium infection (S2B and S2C Fig). A decrease in the protein levels of SQST-1::GFP was restored after iron supplementation. Thus, these results suggest that enhanced susceptibility of worms to *S*. Typhimurium infection by iron is attributable, at least in part, to inhibition of autophagy. Finally, we tested the effect of two non-iron metals, zinc and copper, on survival rate and autophagic activity during *Salmonella* infection. We found that supplementation with CuCl_2_ (100 μM) did not influence survival rate in worms, whereas supplementation with ZnSO_4_ (100 μM) slightly but significantly enhanced sensitivity of worms to *S*. Typhimurium infection (S3A Fig). Unlike FAC, neither ZnSO_4_ nor CuCl_2_ influenced GFP::LGG-1-containing puncta in the seam and intestinal cells after *S*. Typhimurium infection (S3B and S3C Fig). Thus, the inhibitory effect on survival of worms by suppressing autophagy is specific to iron.

### Ferritin mediates host defense against *S*. Typhimurium in the presence of exogenous iron

**Fig 2:**
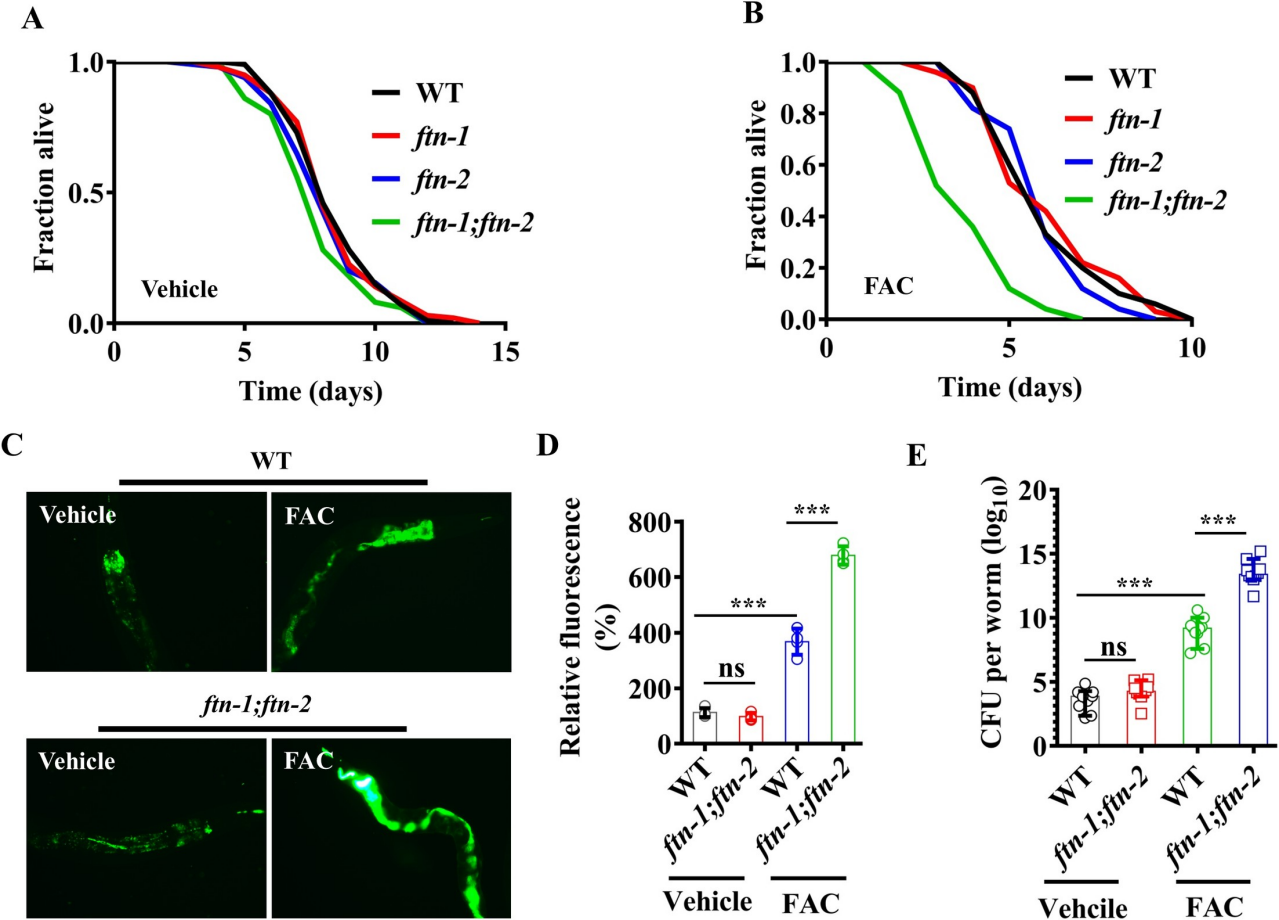
Ferritin is required for defense against *S*. Typhimurium infection in worms in the presence of exogenous iron. **(A and B)** The survival of *ftn-1(ok3625)*, or *ftn-2(ok404)*, or *ftn-1(ok3625);ftn-2(ok404)* double mutants after *S*. Typhimurium infection in the absence (A) or presence (B) of ferric ammonium citrate (FAC, 100 μM). *P*< 0.01, *ftn-1;ftn-2* relative to wild-type (WT) worms in the presence of FAC (Log-rank test). Underlying data are available in S1 Table. **(C)** Representative images of *ftn-1(ok3625);ftn-2(ok404)* double mutants exposed to *S*. Typhimurium expressing GFP for 48 h in the absence or presence of FAC. **(D)** Fluorescence intensity of *S*. Typhimurium expressing GFP. These results are mean ± SD of four independent experiments performed in triplicate (n = 15 worms per experiment). ***P < 0.001; ns, not significant (one-way ANOVA followed by a Student-Newman-Keuls test). Underlying data are available in S2 Table. **(E)** The colony forming units (CFU) of *S*. Typhimurium were measured in worms in the absence or presence of FAC. These results are mean ± SD of 6 independent experiments for the *ftn-1;ftn-2*+FAC group, 10 independent experiments for WT+Vehicle, *ftn-1;ftn-2*+Vehicle, and WT+FAC groups (n ≥ 50 worms per experiment). ***P< 0.001; ns, not significant (one-way ANOVA followed by a Student-Newman-Keuls test). Underlying data are available in S2 Table.

Iron sequestration by the host conferring an innate defense against invading pathogens is termed nutritional immunity [1,31,32]. A previous study has reported that a mutation in *ftn-2* enhances worms’ susceptibility to pathogenic bacteria *E*. *coli* LF82 and *S*. *aureus* [14]. Surprisingly, we found that either *ftn-1(ok3625)* or *ftn-2(ok404)* mutants exhibited a comparable degree of susceptibility to *S*. Typhimurium-mediated killing as WT worms under normal conditions (Fig 2A). Even *ftn-1(ok3625);ftn-2(ok404)* double mutants exhibited similar sensitivity to *S*. Typhimurium infection as did WT worms (Fig 2A). However, *ftn-1(ok3625);ftn-2(ok404)* double mutants, rather than *ftn-1(ok3625)* or *ftn-2(ok404)* single mutants, were more sensitive than WT worms to the killing by *S*. Typhimurium in the presence of 100 μM FAC (Fig 2B). Similar results were confirmed by detecting the survival of worms subjected to *ftn-1* or *ftn-2* RNAi (S4A and S4B Fig). We found that expression of either *ftn-1* or *ftn-2* under its own promoter rescued immune-deficient phenotypes in *ftn-1(ok3625);ftn-2(ok404)* mutants to *S*. Typhimurium infection in the presence of FAC (S5 Fig). Meanwhile, we found that double mutations in *ftn-1;ftn-2* had no impact on the accumulation of *S*. Typhimurium under normal conditions (Fig 2C and 2D). By contrast, the accumulation of *S*. Typhimurium in the intestine of *ftn-1(ok3625);ftn-2(ok404)* double mutants were significantly higher than that in WT worms in the presence of exogenous iron (Fig 2C and 2D). In addition, increased CFU of *S*. Typhimurium were observed in *ftn-1(ok3625); ftn-2(ok404)* double mutants only in the presence of exogenous iron (Fig 2E). These results suggest that *ftn-1* and *ftn-2* are indispensable for defense against *S*. Typhimurium only in the presence of exogenous iron.

Excess iron can be toxic, leading to cell injury and death by initiating oxidative stress [33]. Thus, the observation that *ftn-1(ok3625);ftn-2(ok404)* double mutants exhibited enhanced susceptibility to the killing by *S*. Typhimurium only in the presence of exogenous iron raised a possibility that iron actually induces an abiotic stress response, rather than a biotic stress response, to accelerate worm death. To test this idea, we determined the effect of 100 μM FAC on lifespan, pharyngeal pumping, and defecation rates in worms grown on fed *E*. *coli* OP50. We found that FAC at the concentration did not affect lifespan, pharyngeal pumping, and defecation rates in either WT worms or *ftn-1(ok3625);ftn-2(ok404)* double mutants (S1A–S1C Fig). Finally, we found that the lifespan of *ftn-1(ok3625);ftn-2(ok404)* double mutants grown on heat-killed *S*. Typhimurium in the presence of exogenous iron was comparable of that of these mutants under normal conditions (S2D Fig). These results implicate that 100 μM FAC does not impose any significant stress in ferritin-deficient worms.

### Ferritin confers resistance to *S*. Typhimurium infection in worms when autophagy is inhibited

**Fig 3:**
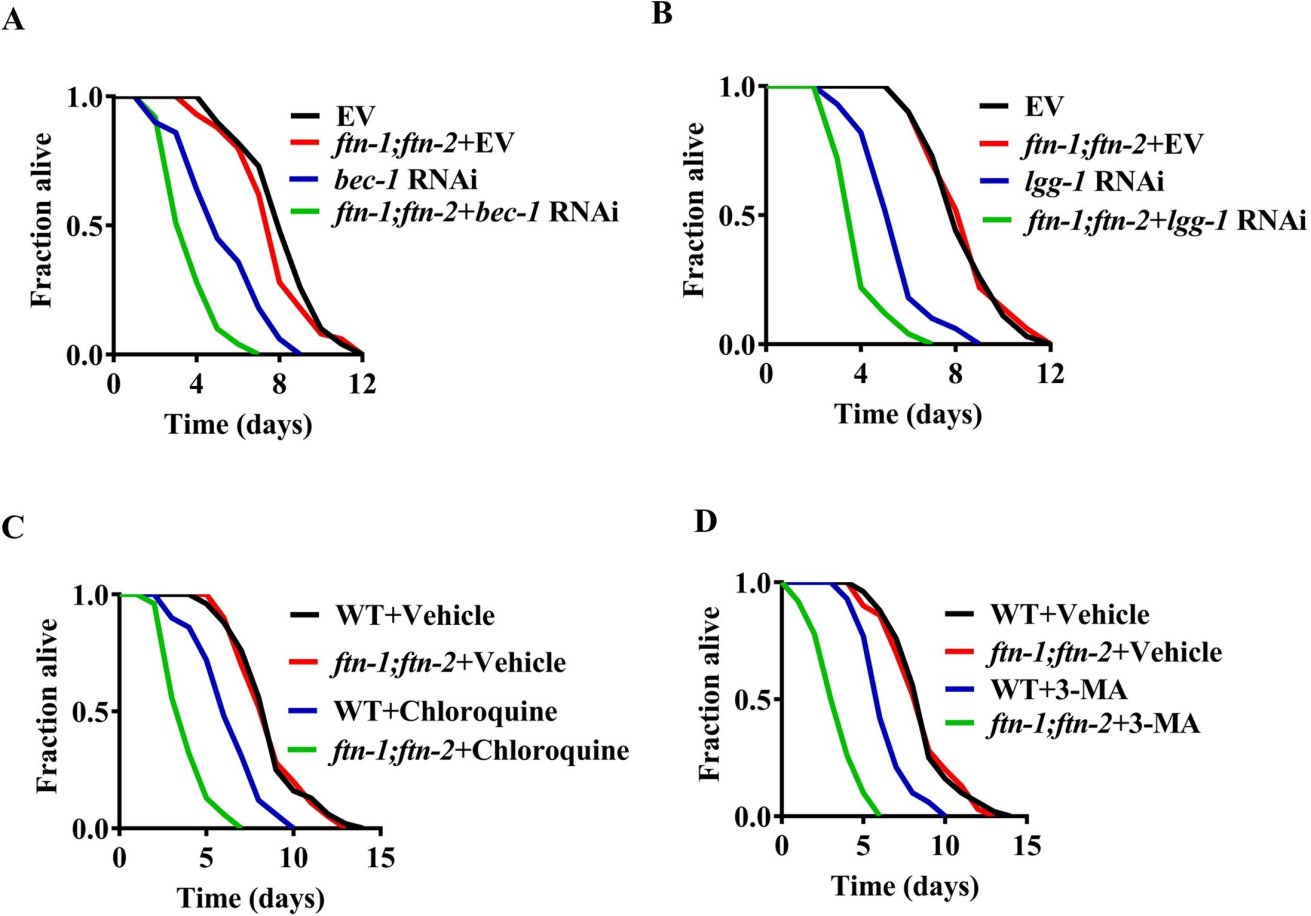
Ferritin is required for defense against *S*. Typhimurium infection after autophagy is inhibited. **(A and B)** *ftn-1(ok3625);ftn-2(ok404)* double mutants were more susceptible to killing by *S*. Typhimurium than wild-type (WT) worms when *bec-1* (A) or *lgg-1* (B) is silenced by RNAi. *P*< 0.01, *bec-1* or *lgg-1* RNAi relative to empty vector (EV); *P*< 0.01, *ftn-1;ftn-2+bec-1* or *lgg-1* RNAi relative to *bec-1* or *lgg-1* RNAi. Underlying data are available in S1 Table. **(C and D)** Chloroquine (C) or 3-methyladenine (3-MA) (D) treatment reduced survival rate of *ftn-1(ok3625);ftn-2(ok404)* double mutants after *S*. Typhimurium infection. *P*< 0.01, WT+Chloroquine or 3-MA relative to WT+Vehicle; *P*< 0.01, *ftn-1;ftn-2+*Chloroquine or 3-MA relative to WT+Chloroquine or 3-MA. *p*-Values throughout were calculated using a Log-rank test. Underlying data are available in S1 Table.

The fact that *ftn-1(ok3625);ftn-2(ok404)* double mutants were more sensitive than WT worms to the killing by *S*. Typhimurium in the presence of exogenous iron implicates that ferritin is involved in host innate defense against infection only under autophagy-deficient conditions. To test this idea, we knockdown two autophagic genes *bec-1* or *lgg-1* by RNAi to inhibit autophagy. In the autophagy-deficient background, *ftn-1(ok3625);ftn-2(ok404)* double mutants were more susceptible than WT worms to the killing by *S*. Typhimurium under normal conditions (Fig 3A and 3B). Next, we treated worms with two inhibitors of autophagy, chloroquine and 3-methyladenine. Likewise, *ftn-1(ok3625);ftn-2(ok404)* double mutants exhibited enhanced susceptibility to *S*. Typhimurium infection in the worms treated with chloroquine or 3-methyladenine (Fig 3C and 3D). These results suggest that ferritin functions as an important host antimicrobial defense when autophagy is inhibited in worms.

### Autophagy mediates degradation of ferritin during *S*. Typhimurium infection

**Fig 4:**
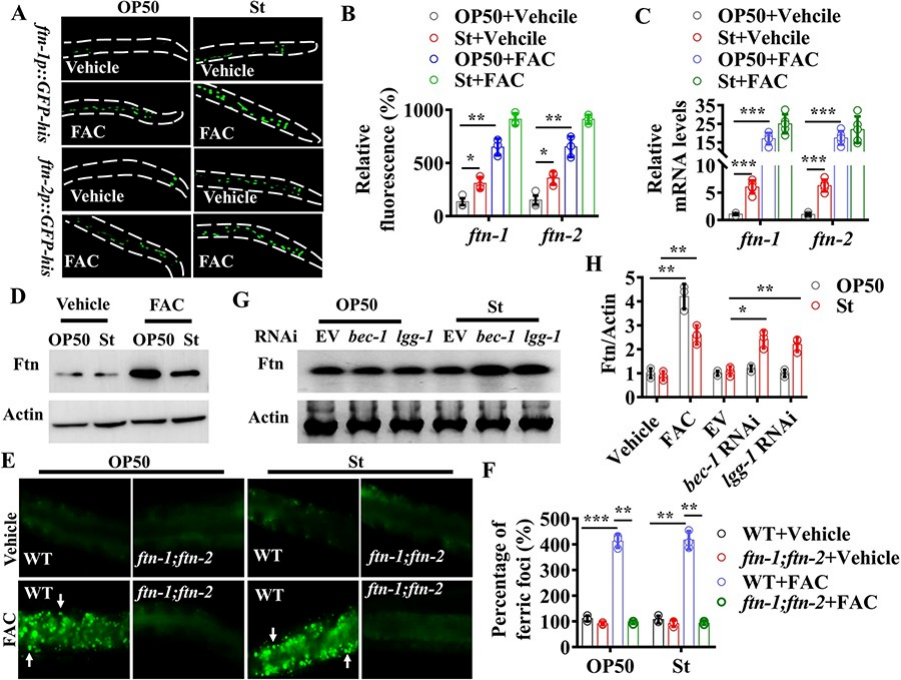
Autophagy promotes degradation of ferritin. **(A)** Expression of *ftn-1p*::*gfp-his* and *ftn-2p*::*gfp-his* was upregulated in worms infected with *S*. Typhimurium (St) or treated with ferric ammonium citrate (FAC, 100 μM) for 12 h. The dotted lines were used to outline the intestine. (**B**) Fluorescence intensity of *ftn-1p*::*gfp-his* and *ftn-2p*::*gfp-his*. These results are mean ± SD of three independent experiments performed in triplicate. (n = 30 worms per experiment). *P < 0.05; **P < 0.01. Underlying data are available in S2 Table. **(C)** The mRNA levels of *ftn-1* and *ftn-2*. These results are mean ± SD of five independent experiments. ***P< 0.001; ns, not significant. Underlying data are available in S2 Table. **(D)** The protein levels of ferritin in worms exposed to *S*. Typhimurium or *E*. *coli* OP50 in the absence or presence of FAC. The blots are typical of three independent experiments. **(E)** Representative images of the ferric foci labelled by RDN3, a ferric (Fe^3+^)-selective fluorescent sensor, in worms exposed to *S*. Typhimurium or *E*. *coli* OP50 for 24 h in the absence (upper panels) or presence (lower panels) of FAC. The arrows denote representative ferric foci. **(F)** Percentage of ferric foci. These results are mean ± SD of three independent experiments. (n = 20 worms per experiment). ***P< 0.001; **P < 0.01. Underlying data are available in S2 Table. **(G)** The protein levels of ferritin in worms subjected to *bec-1* or *lgg-1* RNAi or empty vector (EV) after *S*. Typhimurium infection. The blots are typical of three independent experiments. **(H)** Quantification of ferritin protein levels from Western blot (D and G). These results are mean ± SD of three experiments. *P< 0.05; **P< 0.01. *p*-Values throughout were calculated using a one-way ANOVA followed by a Student-Newman-Keuls test. Underlying data are available in S2 Table.

As mentioned above, *ftn-1(ok3625);ftn-2(ok404)* double mutants exhibited a comparable degree of susceptibility to *S*. Typhimurium infection as WT worms under normal circumstances (Fig 2A). A reasonable explanation is that ferritin expression is inhibited during *S*. Typhimurium infection. Unexpectedly, using the transgenic worms expressing *ftn-1p*::*gfp-his* or *ftn-2p*::*gfp-his*, we observed that the expression of *ftn-1p*::*gfp-his* or *ftn-2p*::*gfp-his* was robustly increased at 12 hours after *S*. Typhimurium infection, compared with that in worms fed *E*. *coli* OP50 (Fig 4A and 4B). qPCR analysis also confirmed that the mRNA levels of *ftn-1* and *ftn-2* were increased in WT worms exposed to *S*. Typhimurium (Fig 4C). It has been shown that exogenous iron activates the transcription of *ftn-1* and *ftn-2* in worms [11]. Consistent with this idea, significant increases in expressions of *ftn-1p*::*gfp-his* and *ftn-2p*::*gfp-his* were observed in worms treated with 100 μM FAC grown on either *E*. *coli* OP50 or *S*. Typhimurium (Fig 4A and 4B). Similar results were confirmed by detecting the mRNA levels of *ftn-1* and *ftn-2* by qPCR (Fig 4C).

Surprisingly, Western blot analysis showed that the protein levels of ferritin were not altered in WT worms after *S*. Typhimurium infection (Fig 4D and 4H). In contrast, iron supplementation significantly upregulated protein levels of ferritin in worms grown on either *E*. *coli* OP50 or *S*. Typhimurium (Fig 4D and 4H). Next, we used RDN3, a ferric (Fe^3+^)-selective fluorescent sensor [34], to label the ferric form. We found that the ferric foci stained by this dye were merged into ferritin, as detected by immunofluorescence (S6 Fig). Thus, the ferric foci mainly represent iron in ferritin. It should be noted that no metal foci were observed in worms after addition of other metals, such as Zn^2+^ and Cu^2+^ (S7A and S7B Fig). We could hardly detect the ferric foci in WT worms grown on *E*. *coli* OP50 (Fig 4E and 4F). As expected, ferric foci were widely observed in WT worms, rather than *ftn-1(ok3625);ftn-2(ok404)* mutants, grown on *E*. *coli* OP50 after addition of 100 μM FAC (Fig 4E and 4F). Furthermore, after *S*. Typhimurium infection, the ferric foci were also detected in WT worms in the presence of exogenous iron (Fig 4E and 4F).

As exogenous iron suppressed *S*. Typhimurium-induced autophagy, these results suggest that autophagy probably mediates degradation of ferritin protein. To test this idea, autophagy was suppressed by *bec-1* or *lgg-1* RNAi. Indeed, Western blot analysis revealed that the protein levels of ferritin in these autophagy-deficient worms were higher than those in worms subjected to empty vector after *S*. Typhimurium infection under normal conditions (Fig 4G and 4H). Furthermore, *bec-1* or *lgg-1* RNAi increased the number of ferric foci in worms infected with *S*. Typhimurium (S8A and S8B Fig). Those observations suggest that although the mRNA of *ftn-1* and *ftn-2* are upregulated by *S*. Typhimurium, the ferritin protein are degraded by autophagy.

### Iron inhibits autophagy by activating TOR after *S*. Typhimurium infection

A previous study has demonstrated that overexpression of *daf-16*, which encodes a forkhead transcription factor, promotes autophagic activity, thus enhancing resistance to *S*. Typhimurium infection in worms [23]. Using the transgenic worms expressing *daf-16p*::*daf-16*::*gfp*, we found that *S*. Typhimurium infection failed to induce nuclear translocation of DAF-16 (S9A Fig). More importantly, knockdown of *daf-16* by RNAi did not influence the abundance of GFP::LGG-1-containing puncta in worms after *S*. Typhimurium infection (S9B Fig). These results implicate that DAF-16 is not involved in the activation of autophagy by *S*. Typhimurium.

**Fig 5:**
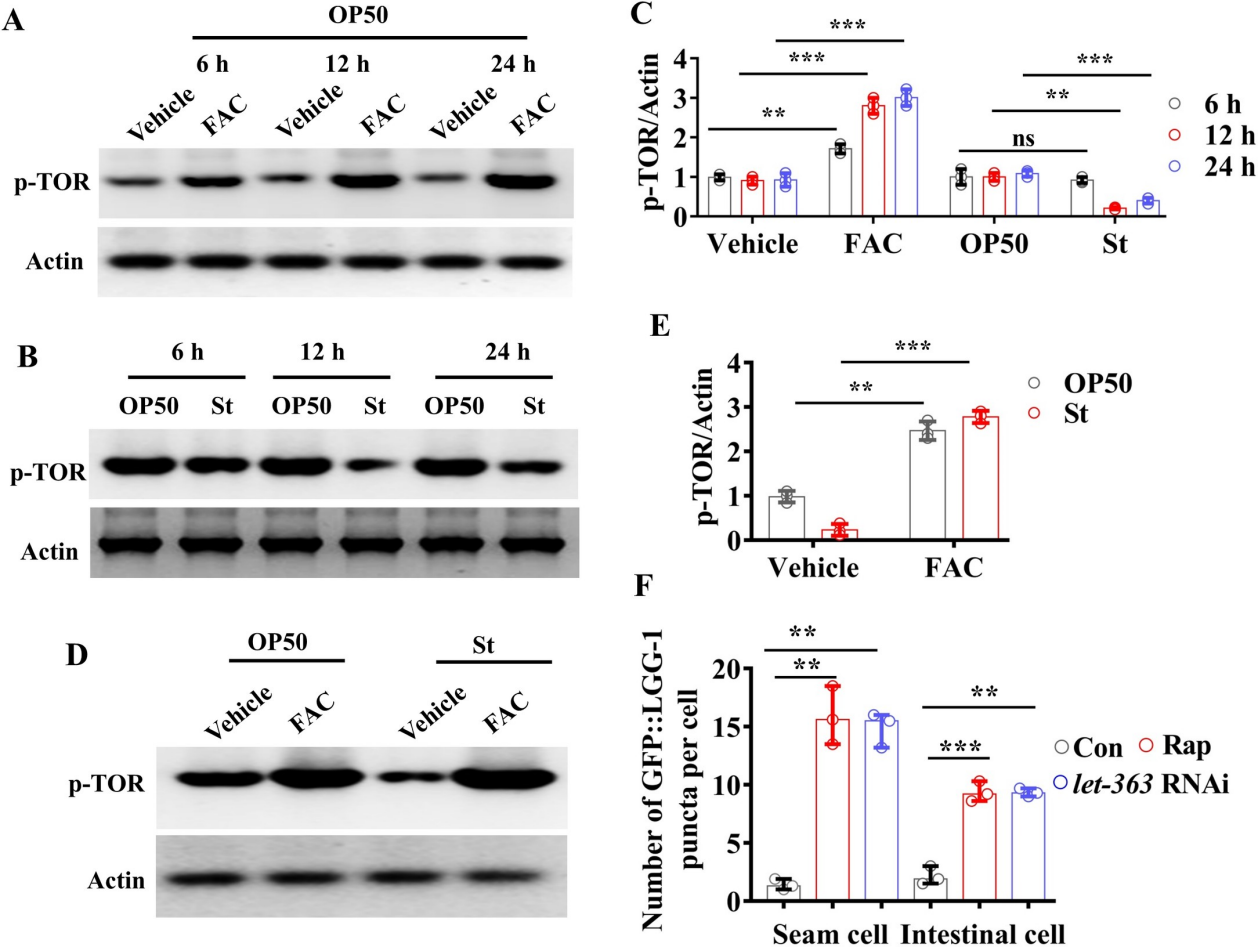
Iron suppresses autophagy via TOR during *S*. Typhimurium infection. **(A)** Supplementation with ferric ammonium citrate (FAC, 100 μM) up-regulated the phosphorylated levels of TOR in wild-type (WT) worms grown on *E*. *coli* OP50. The blots are typical of three independent experiments. **(B)** *S*. Typhimurium (St) infection inhibited the phosphorylated levels of TOR in WT worms. The blots are typical of three independent experiments. **(C)** Quantification of the phosphorylated levels of TOR from Western bot (A and B). These results are mean ± SD of three experiments. **P < 0.01; **P < 0.001; ns, not significant. Underlying data are available in S2 Table. **(D)** The phosphorylated levels of TOR in worms exposed to *S*. Typhimurium or *E*. *coli* OP50 in the absence or presence of FAC. The blots are typical of three independent experiments. **(E)** Quantification of the phosphorylated levels of TOR from Western bot (D). These results are mean ± SD of three experiments. **P < 0.01; **P < 0.001. Underlying data are available in S2 Table. **(F)** *let-363* RNAi or rapamycin (Rap, 0.1 μM) treatment increased autophagy in the seam cells and intestinal cells of worms infected by *S*. Typhimurium in the presence of FAC. The numbers of GFP::LGG-1 puncta were counted. These results are mean ± SD of three independent experiments (n = 15 worms per experiment). **P< 0.01; ***P< 0.001. *p*-Values throughout were calculated using a one-way ANOVA followed by a Student-Newman-Keuls test. Underlying data are available in S2 Table.

Accumulating evidence demonstrates that intracellular iron levels appear to be involved in regulating TOR activity in mammalian cells [35–37]. An iron chelator, desferrioxamine represses TOR activity in mammalian cells [9,37]. In the current study, we found that the levels of phosphorylated TOR were markedly increased in worms grown on *E*. *coli* OP50 at 6, 12 and 24 hours after addition of 100 μM FAC (Fig 5A and 5C). In contrast, *S*. Typhimurium infection led to a significant decrease in the levels of phosphorylated TOR at 12 and 24 hours (Fig 5B and 5C). However, the levels of phosphorylated TOR in worms exposed to *S*. Typhimurium infection were comparable to those in worms grown on *E*. *coli* OP50 in the presence of exogenous iron (Fig 5D and 5E). The phosphorylation levels of TOR were substantially diminished by *let-363/tor* RNAi and by treatment with rapamycin under normal conditions (S10 Fig). As TOR inhibits autophagy in worms [38,39], these results implicate that iron suppresses *S*. Typhimurium infection-induced autophagy by restoring TOR activity. Indeed, inhibition of TOR activity by *let-363/tor* RNAi or rapamycin treatment markedly increased autophagy in worms infected by *S*. Typhimurium in the presence of exogenous iron (Fig 5F).

### TOR controls ferritin proteins after *S*. Typhimurium infection

**Fig 6:**
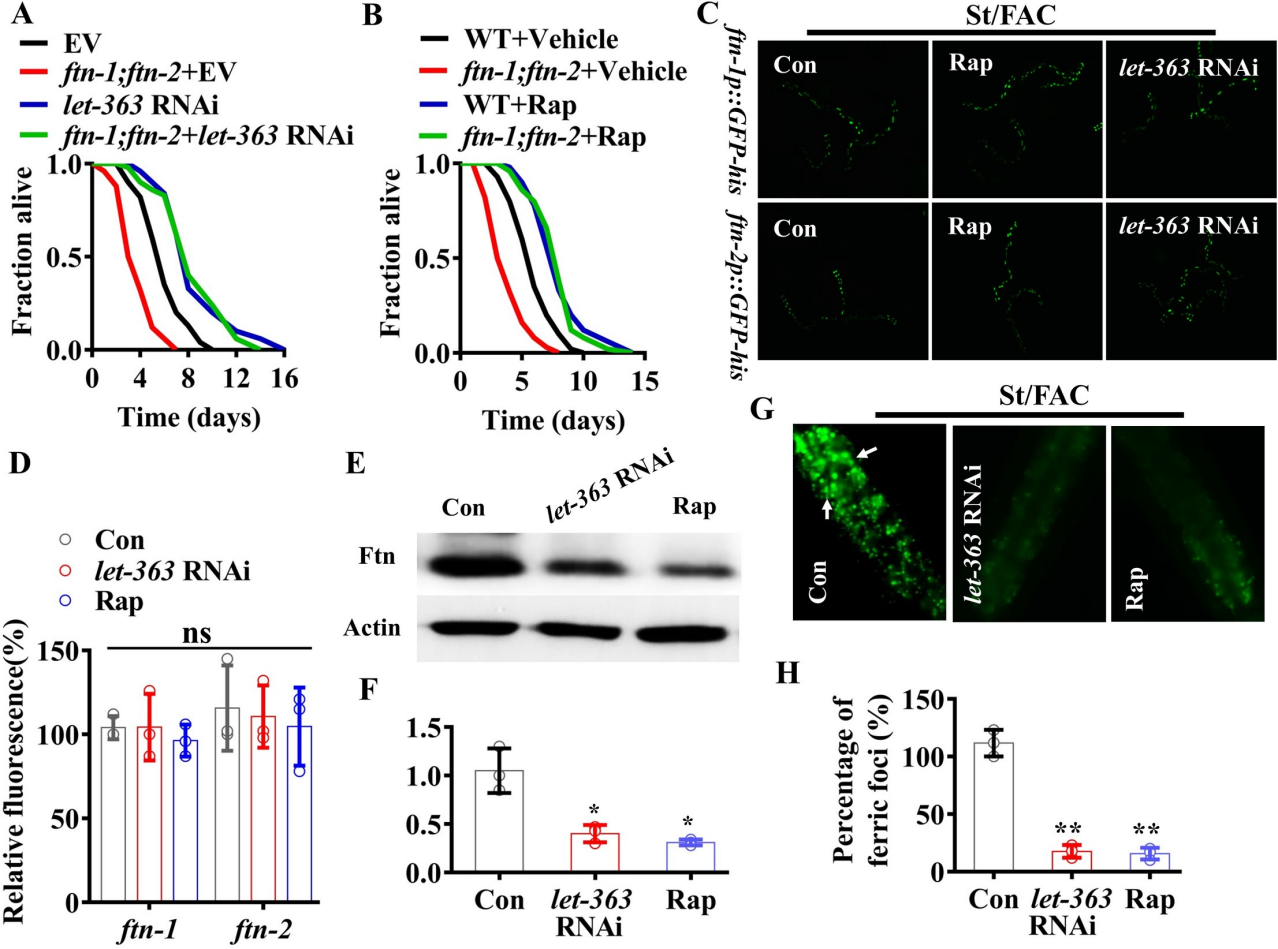
TOR regulates ferritin protein levels during *S*. Typhimurium infection. **(A and B)** *ftn-1(ok3625);ftn-2(ok404)* double mutations did not influence the survival rate of the worms subjected to *let-363* RNAi (A) or rapamycin (Rap) treatment (B) after *S*. Typhimurium infection in the presence of ferric ammonium citrate (FAC, 100 μM) (Log-rank test). Underlying data are available in S1 Table. **(C)** *let-363* RNAi or rapamycin treatment did not alter the expression of *ftn-1p*::*gfp-his* and *ftn-2p*::*gfp-his* in worms infected with *S*. Typhimurium. **(D)** Fluorescence intensity of *ftn-1p*::*gfp-his* and *ftn-2p*::*gfp-his*. These results are mean ± SD of three independent experiments performed in triplicate. (n = 30 worms per experiment). ns, not significant (one-way ANOVA followed by a Student-Newman-Keuls test). Underlying data are available in S2 Table. **(E)** *let-363* RNAi or rapamycin treatment suppressed the levels of ferritin protein in worms infected with *S*. Typhimurium in the presence of FAC. The blots are typical of three independent experiments. Con, control. **(F)** Quantification of ferritin protein levels from Western blot (E). These results are mean ± SD of three experiments. *P < 0.05 (one-way ANOVA followed by a Student-Newman-Keuls test). Underlying data are available in S2 Table. **(G)** Representative images of ferric foci in worms subjected to *let-363* RNAi infected with *S*. Typhimurium in the presence of FAC. The arrows denote representative ferric foci. **(H)** Percentage of ferric foci. These results are mean ± SD of three independent experiments. (n = 20 worms per experiment). **P < 0.01 (one-way ANOVA followed by a Student-Newman-Keuls test). Underlying data are available in S2 Table.

In this study, we found that after *S*. Typhimurium infection, the survival rate of *ftn-1(ok3625);ftn-2(ok404)* double mutants subjected to *let-363/tor* RNAi or rapamycin treatment was comparable to that of WT worms subjected to *let-363/tor* RNAi or rapamycin treatment in the presence of 100 μM FAC (Fig 6A and 6B). We thus determined whether TOR was involved in regulation of ferritin by iron during *S*. Typhimurium infection. In the presence of exogenous iron, we found that *let-363/tor* RNAi or rapamycin treatment did not alter the expression of *ftn-1p*::*gfp-his* and *ftn-2p*::*gfp-his* in worms infected with *S*. Typhimurium (Fig 6C and 6D). Similar results were confirmed by detecting the mRNA of *ftn-1* and *ftn-2* levels using qPCR (S11 Fig). In contrast, Western blot analysis revealed that *let-363/tor* RNAi or rapamycin treatment significantly suppressed the levels of ferritin protein during *S*. Typhimurium infection in the presence of exogenous iron (Fig 6E and 6F). Furthermore, *let-363/tor* RNAi or rapamycin treatment reduced the number of ferric foci in worms after *S*. Typhimurium infection, compared with worms subjected to empty vector in the presence of exogenous iron (Fig 6G and 6H). Taken together, our findings suggest that iron modulates the protein levels of ferritin via TOR.

## Discussion

**Fig 7:**
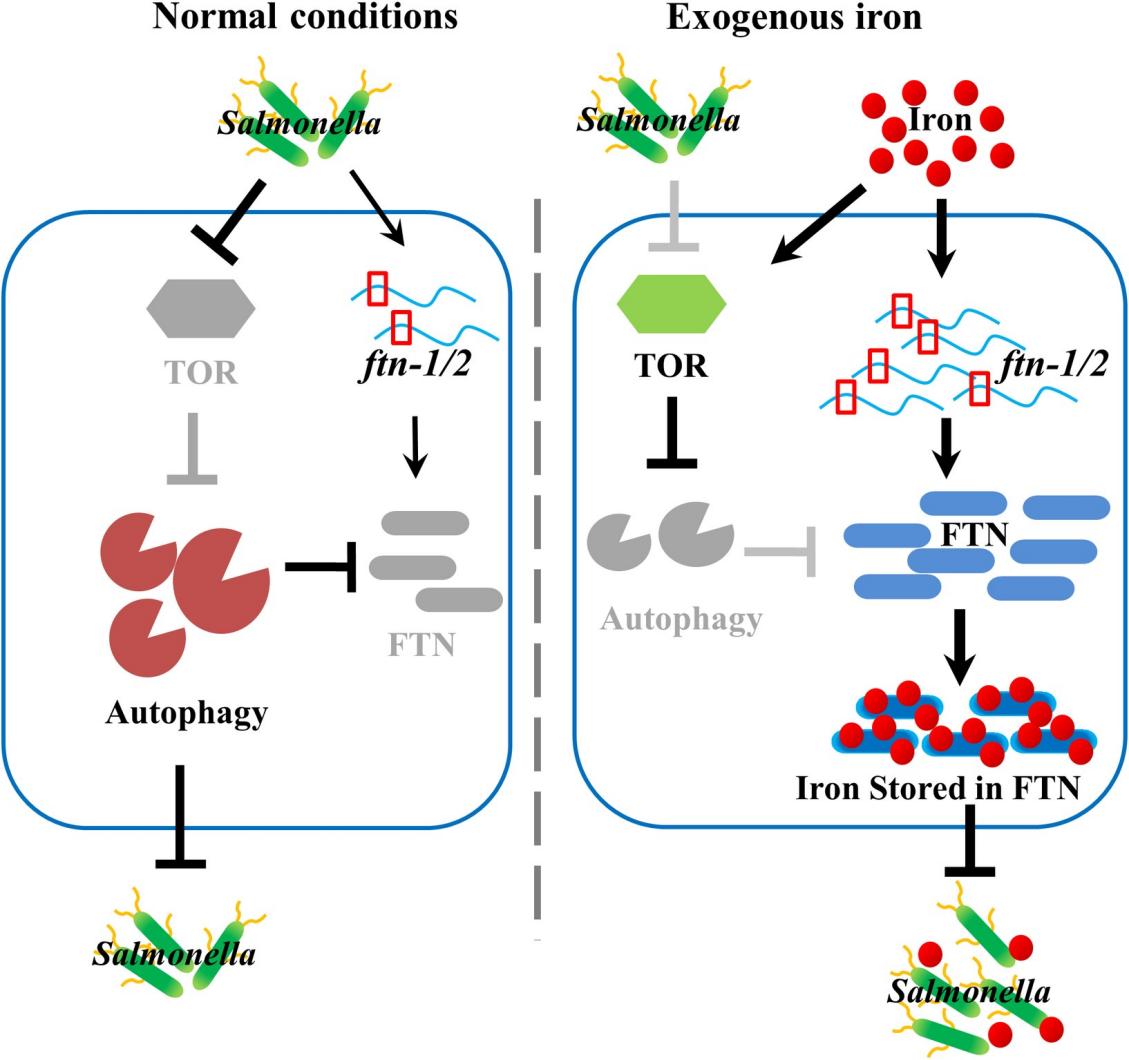
Schematic model for TOR-mediated innate immunity. As an iron sensor, Left panel: Under normal circumstances, TOR is inhibited upon *Salmonella* infection, leading to activation of autophagy and degradation of ferritin protein. Autophagy is responsible for inhibiting this pathogen by an unknown mechanism. Right panel: Exogenous iron activates TOR resulting in inhibition of autophagy and up-regulation of ferritin protein levels. Ferritin sequesters iron, an essential nutrient for *Salmonella*, to limit its proliferation.

Our findings demonstrate that TOR could function as a molecular switch connecting an iron cue to defend against pathogen infection in *C*. *elegans* (Fig 7). Under normal conditions, TOR is inactivated by *S*. Typhimurium infection to promote autophagy, which is essential for worms to defend against *Salmonella*. When exogenous iron exists, TOR is activated to suppress autophagy, leading to increased ferritin levels. Ferritin may limit bacterial growth by reducing iron availability. Thus, our study unmasks a previously unsuspected role of TOR in regulating innate immune responses to bacterial infection via sensing iron levels.

Autophagy has been shown to play an important role in defense against *S*. Typhimurium infection in worms [23,26]. However, how *S*. Typhimurium infection activates autophagy is unknown. Although overexpression of *daf-16* promotes autophagic activity in worms [23], our data demonstrated that *daf-16* was not involved in *S*. Typhimurium-induced activation of autophagy. In addition, it has been shown that a mutation in *daf-16(m26)* does not significantly affect *S*. Typhimurium colonization in the gut of worms [40]. These results suggest that *daf-16* is not required for resistance to *S*. Typhimurium infection. In this study, we found that *S*. Typhimurium infection suppressed the activity of TOR, a negative regulator of autophagy [38,39]. A decrease in TOR activity is also observed in macrophages infected with a pathogenic bacterium *Legionella pneumophila* [41]. Thus, inhibition of TOR leads to the activation of autophagy upon *S*. Typhimurium infection. Furthermore, our study demonstrated that iron supplementation inhibited autophagy, thereby accelerating worm death by *S*. Typhimurium infection. Previous studies have demonstrated that iron depletion inhibits the TOR activity in mammalian cells [9,35–37]. Consistent with these observations, we found that supplementation with iron markedly increased the TOR activity in worms. Thus, iron may inhibit autophagy, an important component of host defense system, by activating the TOR signaling.

Autophagy can limit the levels of intracellular pathogens by targeting these microbes for degradation in cells [42]. Although Salmonella is a facultative intracellular pathogen that can be found in a variety of mammalian cells, the bacterium appears to remain predominantly in the intestinal lumen of worms upon infection [26]. How does autophagy provide defense against Salmonella infection in worms? Previously, Visvikis et al. have demonstrated that HLH-30, the C. elegans ortholog of TFEB-1, mediates a transcriptional response to *S. aureus*, including a set of anti-microbial and autophagy genes, thereby providing defense and tolerance to infection [25]. Our previous study also indicates that induction of autophagy prevents necrosis, and protects worms against Pseudomonas aeruginosa infection [24]. In addition, mitophagy is responsible for organismal resistance to pyoverdin, a virulence factor that can kill worms [27]. Likewise, autophagy promotes tolerance to *Bacillus thuringiensis* infection via degradation of pore-forming toxin Cry5B secreted by this pathogen and repair of damaged membrane in worms [43]. Based on these observations, it is reasonable to speculate that autophagy may confer resistance against Salmonella infection by repairing organismal insults.

Nutritional immunity is a process by which animals sequesters transitional metals, such as iron, to limit pathogenicity during infection [1,31,32]. Ferritin is believed to enhance the ability of host to compete with bacteria for iron, thereby inhibiting bacterial proliferation. For example, FTN-2 protein levels are up-regulated by a human pathogen *E*. *coli* LF82 and confers worm resistance to the killing by this pathogen as well as *S*. *aureus* under normal conditions [14]. However, we found that either a single mutation in *ftn-1* or *ftn-2* or double mutations in *ftn-1;ftn-2* did not alter susceptibility to the killing by *S*. Typhimurium in worms under normal conditions. In contrast, *ftn-1(ok3625);ftn-2(ok404)* double mutants, but not *ftn-1(ok3625)* or *ftn-2(ok404)* single mutants, were more sensitive to *S*. Typhimurium infection than WT worms in the presence of exogenous iron. Meanwhile, expression of either *ftn-1* or *ftn-2* was sufficient to rescue immune-deficient phenotypes in *ftn-1(ok3625); ftn-2(ok404)* mutants to *S*. Typhimurium infection. These results suggest that *ftn-1* and *ftn-2* are functionally redundant. In this study, we found that although the mRNA levels of *ftn-1* and *ftn-2* were significantly up-regulated, the protein levels of ferritin were not altered by *S*. Typhimurium infection under normal conditions. In general, a variety of factors, such as post-transcriptional or post-translational regulation, and different rates of protein turnover, may account for the discrepancy between transcript and protein levels [44,45]. In mammals, nuclear receptor coactivator 4 (NCOA4) together with the autophagic protein ATG8 delivers ferritin to the lysosome for recycling, a process termed ferritinophagy [46–48]. Although there is no homology for NCOA4 in *C*. *elegans*, our results demonstrated that autophagy was responsible for the degradation of ferritin induced by *S*. Typhimurium. In the presence of exogenous iron, the protein level of ferritin was markedly increased during *S*. Typhimurium infection, compared with those under normal conditions. The increase in ferritin protein is probably achieved in two ways: one is to up-regulate the transcription of *ftn-1* and *ftn-2* and the other is to reduce the degradation of ferritin by inhibiting autophagy. In addition, we found that *ftn-1* and *ftn-2* were responsible for defense against *S*. Typhimurium infection in the presence of exogenous iron, rather than under normal conditions. Thus, whether ferritin is involved in innate immunity depends on its protein levels in worms.

Ferritin is important for iron homeostasis. A previous study from Kim et al. has shown that supplementation with 13.52 mM FAC significantly reduces lifespan in *ftn-1* (RNAi) worms, but not *ftn-2 (ok404)* mutants, grown on *E*. *coli* OP50 [49]. In the current study, double mutations in *ftn-1;ftn-2* reduced the survival of worms upon *S*. Typhimurium infection only in the presence of exogenous iron. The role of ferritin may be to sequester iron to protect the organism from oxidative damage caused by iron, rather than organismal insults induced by pathogens. However, our data presented here demonstrated that as little as 100 μM of added FAC reduced the survival of worms upon *S*. Typhimurium infection in *ftn-1;ftn-2* double mutants. Under such a concentration, FAC did not significantly influence various traits, including lifespan, in *ftn-1;ftn-2* double mutants under non-pathogen conditions. Actually, even supplementation with 9 mM FAC does not reduce the lifespan of WT worms grown on *E*. *coli* OP50 [4]. Thus, our data exclude a direct causal role for exogenous iron in reduced survival of worms by inducing an abiotic stress response.

TOR is a crucial sensor of energy state and a fundamental hub for integration of environmental stimuli and nutritional stress [9,50]. Our data reveal that activation of autophagy and induction of ferritin represent two facets of host innate defense. Under normal conditions, autophagy promotes tolerance to infection. When exogenous iron exists, ferritin acts to limit the availability of free iron for pathogen. However, the two lines of defense may not work simultaneously. Activation of autophagy leads to degradation of ferritin, whereas induction of ferritin by exogenous iron is accompanied by inhibition of autophagy. Our findings reveal that TOR functions as a molecular switch determining which one state may involve host defense in response to a nutritional cue, iron. The fact that the TOR signaling known to sense nutritional cues coordinates immune responses, reflecting functional integration of nutrient- and pathogen-sensing pathways. Understanding of the physiological interplay between nutritional and immune responses may provide insight into the basis for the treatment of infectious diseases.

## Materials and methods

### Nematode strains

WT worms were *C*. *elegans* Bristol N2 strain. Mutated and transgenic strains used in this study include the following: *ftn-1(ok3625)*, *ftn-2(ok404)*, XA6900 (*pha-1(e2123)*; qaEx6900 *[ftn-1p*::*pes-10*::*GFP-his+pha-1(+)]*), XA6901 (*lin-15B& lin-15A(n765)*; qaEx6901[*ftn-2p*::*pes-10*::*GFP*::*his+lin-15(+)]*), and *lgg-1p*::*gfp*::*lgg-1* kindly provided by the Caenorhabditis Genetics Center (CGC; http://www.cbs. umn.edu/CGC), which is funded by NIH Office of Research Infrastructure Programs (P40 OD010440). Mutants were backcrossed three times into the N2 strain used in our laboratory. All strains were grown on nematode growth media (NGM) and fed on *E*. *coli* OP50 at the temperature of 20°C [51].

### Infection of worms with bacteria

For survival assays of worms, synchronized L1 larvae were cultivated fed *E*. *coli* OP50 at 20°C until the young adult stage. *S*. *enterica* serovar Typhimurium 468 (a gift from Dr. WH Lee, Kunming Institute of Zoology, CAS) were seeded on the killing assay plates, which contained modified NGM (0.35% instead of 0.25% peptone) and 75 μg/ml of 5’-fluoro-2’-deoxyuridine (FUdR) [45,52]. The infection experiments were started by adding 50–60 nematodes to each plate at 25°C. The number of living animals was counted at time intervals. Three killing assay plates were tested per assay and all experiments were performed three times independently.

### RNA interference

RNAi bacterial strains containing the vector of expressing dsRNA of targeting genes were obtained from the Ahringer RNAi library [53]. Firstly, RNAi strains of *E*. *coli* HT115 (DE3) was grown in LB broth with 100 μg/ml ampicillin (Sangon Biotech Co., Shanghai, China) at 37°C for 8–12 hours. Then 200–400 μl of the bacterial suspension was dispensed onto NGM plates containing 100 μg/ml ampicillin (Sangon Biotech Co.) and 5 mM isopropyl 1-thio-β-D-galactopyranoside (IPTG) (Sangon Biotech Co.). These RNAi-expressing bacteria were allowed to grow for 12–16 hours at 25°C. Then L1 larvae of worms were placed on RNAi NGM plates at 20°C until they reached maturity. Young adult worms were used for further assays.

### Quantitative RT-PCR

Total RNA was extracted from worms with TRIzol Reagent (Invitrogen, Carlsbad, CA). Random-primed cDNAs were generated by reverse transcription of the total RNA samples with SuperScript II (Invitrogen). A quantitative real-time PCR analysis was performed using SYBR Premix (Takara, Dalian, China) on a Roche LightCycler 480 System (Roche Applied Science, Penzberg, Germany). The relative amount of *ftn-1* or *ftn-2* mRNA to *ama-1* mRNA (an internal control) was calculated using the method described previously [11,54]. The primers used for PCR were as follows: *ama-1*: 5’- TCC TGA CCC AAA GAA CAC GGT-3’ (F), 5’-ATC CAC CTG CTC CTC CTG AG-3’ (R); *ftn-1*: 5’-GAA TTC AAG CTG TCC GTG G-3’ (F), 5’-CGA ATG TAC CTG CTC TTC C-3’ (R); *ftn-2*: 5’-GTT AAC AAG CAG ATC AAC ATT-3’ (F), 5’-CAT TCT CTG GCT TCT GGA TG-3’ (R).

### Protein extraction and immunoblotting

After washed with M9 buffer, worms were immediately homogenized in liquid nitrogen. Then the homogenate was lysed on ice for 30 minutes in lysis buffer RIPA (Beyotime Institute of Biotechnology, Haimen, China). The supernatant was obtained from cell lysates by centrifugation at 12,000 rpm for 15 minutes at 4°C, and used for Western blot analysis. The proteins of lysates were loaded (50 μg per well) were subjected to SDS-polyacrylamide gel electrophoresis. The anti-ferritin rabbit polyclonal antibodies obtained from Abmart Inc. (Shanghai, China) was raised against peptide RDDIALRNIAKF. The other primary antibodies were anti-Actin antibodies (ACTN05, mouse mAb, 1:1000 dilution; Abcam), anti-Phospho-mTOR (Ser2448) (5536, rabbit polyclonal antibody, 1:1000 dilution; Cell Signaling Technology), and anti-GFP antibodies (1:1000 dilution; Abmart Inc.). The secondary antibodies used were HRP-conjugated anti-mouse (1:5000 dilution; Beijing TransGen Biotech Co.) or rabbit IgG (1:5000 dilution; Abmart Inc.). An imaging system (Amersham Imager 600) was used for documentation of the Western blot results.

### Microscopy

For detecting fluorescent signals in worms, *ftn-1p*::*pes-10*::*gfp-his* and *ftn-2p*::*pes-10*::*gfp-his* strains were mounted in M9 onto microscope slides. Ferric (Fe^3+^)-selective fluorescent sensor RDN3 were used to stain label the ferric form stored in worms according to the method described previously [34]. The worms were analyzed using a Zeiss Axioskop 2 Plus fluorescence microscope (Carl Zeiss, Jena, Germany). At least 30 worms were examined per assay in three or four independent experiments.

### Autophagy analysis

Synchronized populations of worms were cultivated at 20°C until they reached the young adult stage. After infected with *S*. Typhimurium or treated with FAC, the worms were mounted in M9 onto microscope slides. Transgenic worms expressing GFP::LGG-1 were injected with 50 mM BafA or DMSO. Two hours after injection, these worms were immediately mounted in M9 onto microscope slides. The slides were viewed using a Zeiss Axioskop 2 plus fluorescence microscope (Carl Zeiss, Jena, Germany). For worms carrying GFP::LGG-1, GFP::LGG-1 positive puncta were counted in either the seam cells or the intestine. At least 15 worms were examined per assay in three independent experiments.

### Detection of bacterial accumulation in worms

Colony forming units (CFU) of *S*. Typhimurium in the intestine of worms were performed as described previously [55]. For measurement of *S*. Typhimurium CFU, mild-L4 worms were exposed to NGM plates with *S*. Typhimurium expressing GFP (the vector for GFP expression is a gift from Dr. XP Qi, Kunming Institute of Zoology, CAS) for 48 hours at 25°C. Then worms were collected and soaked in M9 buffer containing 25 mM levamisole hydrochloride (Sangon Biotech Co.) and 100 μg/ml ampicillin (Sangon Biotech Co.) for 30 minutes at room temperature. Then worms were washed three times with M9 buffer. Some of worms were immediately mounted in M9 onto microscope slides. The slides were viewed using a Zeiss Axioskop 2 plus fluorescence microscope (Carl Zeiss, Jena, Germany) with a digital camera. At least 15 worms were examined per assay in four independent experiments. Meanwhile, about 50 of nematodes per group were transferred into 50 μl PBS plus 0.1% Triton and ground. The lysates were diluted by 10-fold serial dilutions in sterilized water and spread over Chromogenic/Fluorogenic Culture Media agar (Sangon Biotech Co.) plates with 100 μg/ml ampicillin. After incubation overnight at 37°C, the colonies of GFP-*S*. Typhimurium were counted. For each group, 6–10 independent experiments were carried out.

### Phenotype assays

Synchronized worms were cultivated on NGM agar plates at of 20°C until they reached the late L4 stage, and then shifted to two new NGM agar plates. These plates containing *E*. *coli* OP50 or *S*. Typhimurium in the presence or absence of 100 μM FAC, respectively. (1) Lifespan analysis. The experiment was performed as previously described [30]. The first day of adulthood was recorded as day 1. During their reproductive period, these worms were transferred to new plates every day. After reproduction ceased, worms were transferred to new plate every third day. The number of worms was scored every day. If worms did not move when gently prodded and displayed no pharyngeal pumping, they were marked as dead. Three plates of 50–60 animals per plate were tested per assay and all experiments were performed three times independently. (2) Pharyngeal pumping assay. Pharyngeal pumping was monitored by counting the number of contractions in the terminal bulb of pharynx in 30 s intervals. At least 30 worms were examined per assay in three independent experiments. (3) Defecation rate assay. The experiment was performed as previously described [56]. In briefly, a defecation cycle was defined as the duration time between the pBoc steps of two consecutive defecations. Each worm was scored for five consecutive cycles. 20–30 worms were examined per assay in three independent experiments.

### Construction of transgenes

To make construct of *ftn-1p*::*ftn-1*::*mCherry* and *ftn-2p*::*ftn-2*::*mCherry*, a 4048 bp fragment of *ftn-1* gene and a 3580 bp fragment of *ftn-2* gene from genomic DNA were chemically synthesized (Generay Biotech Co., Shanghai, China). These fragments were inserted into the pCFJ104 [57] by replacing the promoter myo-3 with *ftn-1p*::*ftn-1* and *ftn-2p*::*ftn-2*, respectively. Twenty-five ng/ml DNA fragments of either *ftn-1p*::*ftn-1*::*mCherry* or *ftn-2p*::*ftn-2*::*mCherry* were injected into the syncytial gonads of *ftn-1(ok3625);ftn-2(ok404)* double mutants with 50 ng/ml pRF4 as a transformation marker [58]. The transgenic worms carrying *ftn-1p*::*ftn-1*::*mCherry* or *ftn-2p*::*ftn-2*::*mCherry* were confirmed in prior to pathogenesis assay.

### Immunofluorescence staining

After infected with *S*. Typhimurium or treated with FAC, worms were washed three times with M9 buffer and mounted in M9 onto microscope slides. Then the worms were fixed with 1× MRWB buffer (2× MRWB buffer contains 160 mM KCl, 40 mM NaCl, 14 mM Na_2_EGTA, 1 mM spermidine-HCl, 0.4 mM spermine, 30 mM Na-PIPES, and 0.2% ß-mercaptoethanol, pH 7.4) containing 4% paraformaldehyde (PFA) for 1 hour at room temperature. After washed three times by M9 buffer, worms were fixed in ice-cold methanol for 5  minutes at room temperature. Subsequently, the worms were blocked with the mixture of 10% BSA for 1 hour at room temperature. Following the blocking, the worms were incubated with primary antibodies specific for ferritin (rabbit polyclonal antibodies; 1:150 dilution; Abmart Inc) overnight at 4°C. After washed three times in PBS with 0.1% Tween-20 (PBST), the worms were incubated with goat anti-rabbit IgG (dilution 1:50, Jackson ImmunoResearch Laboratories, West Grove, PA) for 2 hours. After washed three times with PBST, the worms were stained with 1 μg/ml of 4,6-diamidino-2-phenylindole (DAPI) for 15 minutes to detect nuclei. After stained with DAPI, the worms transfer to slides, were viewed using on a Zeiss Axioskop 2 Plus fluorescence microscope.

### Detection of ferric iron

After infected with *S*. Typhimurium or treated with FAC, worms were washed three times with M9 buffer and mounted in M9 onto microscope slides. Then the worms were fixed with M9 buffer containing 4% paraformaldehyde for 30 minutes at room temperature. Following the fixing, the worms were immediately frozen in liquid nitrogen and subjected to one freeze/thaw cycles. After washed with M9 buffer, the worms were dehydrated in 50 μM RDN3 for 3 hours. After washed three times with M9 buffer, the slides were viewed using on a Zeiss Axioskop 2 Plus fluorescence microscope. The ferric foci were counted in the intestine of worms. At least 20 worms were examined per assay in three independent experiments.

### Drug treatment

Standard killing assay plates seeded with *E*. *coil* OP50 or *S*. Typhimurium were supplemented with 30 mM chloroquine (S4157, Selleckchem, Shanghai, China), or 100 mM 3-methyladenine (S2767, Selleckchem) or 100 nM Rapamycin (S1039, Selleckchem). ddH_2_O or DMSO was used as a control.

### Statistics

Differences in survival rates were analyzed using the log-rank test. Differences in mRNA and protein levels, the number of GFP::LGG-1 positive puncta, the number of ferric foci, pharyngeal pumping and defecation rate, and CFU were assessed by performing a one-way ANOVA followed by a Student-Newman-Keuls test. Data were analyzed using GraphPad Prism 7.0.

## Supporting information

S1 Fig FAC does not impose significant stresses in ferritin-deficient worms. (A and B) FAC did not affect pharyngeal pumping and defecation rates in either WT worms or *ftn-1(ok3625);ftn-2(ok404)* double mutants grown on fed *E*. *coli* OP50 or exposed to *S*. Typhimurium (St). These results are mean ± SD of three independent experiments. ns, not significant (one-way ANOVA followed by a Student-Newman-Keuls test). Underlying data are available in S2 Table. (C) Ferric ammonium citrate (FAC, 100 μM) did not affect lifespan in either WT worms or *ftn-1(ok3625);ftn-2(ok404)* double mutants grown on fed *E*. *coli* OP50 (Log-rank test). Underlying data are available in S1 Table. (D) Lifespan of *ftn-1(ok3625);ftn-2(ok404)* double mutants grown on heat-killed *S*. Typhimurium (HK-St) in the presence of FAC was comparable to that of these mutants under normal conditions (Log-rank test). Underlying data are available in S1 Table.
(TIF)

S2 Fig *S*. Typhimurium promotes autophagic flux. (A) The numbers of GFP::LGG-1 puncta were counted in the seam cells (left panel) and intestinal cells (right panel) of worms infected by *S*. Typhimurium in the absence or presence of ferric ammonium citrate (100 μM). FAC. After infected with *S*. Typhimurium or treated with FAC, the transgenic worms expressing GFP::LGG-1 were injected with 50 mM BafA or DMSO. These results are mean ± SD of three independent experiments (n = 15 worms per experiment). *P< 0.05; **P< 0.01. Underlying data are available in S2 Table. (B) The levels of SQST-1::GFP were measured by Western blot. The blot shown here is typical of three independent experiments. (C) Quantification SQST-1::GFP from Western blot (B). These results are mean ± SD of three independent experiments. **P< 0.01; ***P< 0.001. *p*-Values throughout were calculated using a one-way ANOVA followed by a Student-Newman-Keuls test. Underlying data are available in S2 Table.
(TIF)

S3 Fig Effects of ZnSO_4_ and CuCl_2_ on survival and autophagic activity in worms. (A) Supplementation with CuCl_2_ (100 μM) did not influence survival rate (*P*> 0.05 vs vehicle), whereas supplementation with ZnSO_4_ (100 μM) slightly but significantly enhanced sensitivity of worms after *S*. Typhimurium infection (*P*< 0.05, ZnSO_4_ vs vehicle). *p*-Values throughout were calculated using a Log-rank test. Underlying data are available in S1 Table. (B and C) The numbers of GFP::LGG-1 puncta were counted in the seam cells (B) and intestinal cells (C) of worms exposed to *S*. Typhimurium for 12 h. These results are mean ± SD of three independent experiments (n = 15 worms per experiment). ns, not significant (one-way ANOVA followed by a Student-Newman-Keuls test). Underlying data are available in S2 Table.
(TIF)

S4 Fig Knockdown of *ftn-1* and *ftn-2* are more susceptible to *S*. Typhimurium infection during iron overload. (A and B) The survival rates of worms subjected to *ftn-1* RNAi, *ftn-2* RNAi, or *ftn-1*;*ftn-2* RNAi in the absence (A) or presence (B) of 100 μM ferric ammonium citrate (FAC, 100 μM) after *S*. Typhimurium infection. *P*< 0.01, *ftn-1*;*ftn-2* RNAi relative to empty vector (EV) (B). *p*-Values throughout were calculated using a Log-rank test. Underlying data are available in S1 Table.
(TIF)

S5 Fig Expression of either *ftn-1* or *ftn-2* rescues immune-deficient phenotypes in *ftn-1(ok3625);ftn-2(ok404)* mutants to *S*. Typhimurium infection in the presence of exogenous iron. The concentration of ferric ammonium citrate (FAC) was 100 μM. *P*< 0.01 relative to *ftn-1;ftn-2* (Log-rank test). Underlying data are available in S1 Table.
(TIF)

S6 Fig Ferric foci represents iron in ferritin. The ferric foci stained by RDN3, a ferric (Fe^3+^)-selective fluorescent sensor, were merged into ferritin detected by immunofluorescence. (A) Immunofluorescence with anti-Ftn antibodies; (B) RDN3 staining; (C) DAPI staining; (D) DIC image; (E and F) Enlarged views were shown for areas. (G) Merged image. The arrows denote representative ferric foci.
(TIF)

S7 Fig No metal foci are observed in worms after addition of Zn^2+^ and Cu^2+^. (A) Worms were stained with RDN3 in the presence of Zn^2+^ and Cu^2+^. (B) Autofluorescence in worms without RDN3 under normal conditions.
(TIF)

S8 Fig Inhibition of autophagy increases the number of ferric foci in worms infected with *S*. Typhimurium. (A) Worms were stained by RDN3 staining upon *S*. Typhimurium infection. The arrows denote representative ferric foci. (B) Percentage of ferric foci. These results are mean ± SD of three independent experiments. *P< 0.05; **P< 0.01 relative to empty vector (EV) (one-way ANOVA followed by a Student-Newman-Keuls test). Underlying data are available in S2 Table.
(TIF)

S9 Fig DAF-16 is not involved in the activation of autophagy by *S*. Typhimurium. (A) *S*. Typhimurium (St) infection did not induce nuclear translocation of DAF-16. (B) Knockdown of *daf-16* by RNAi did not influence autophagy in worms after *S*. Typhimurium infection. The numbers of GFP::LGG-1 puncta were counted in the seam cells and intestinal cells of worms. These results are mean ± SD of three independent experiments (n = 15 worms per experiment). ns, not significant (two sample t-test). Underlying data are available in S2 Table.
(TIF)

S10 Fig *let-363/tor* RNAi and treatment with rapamycin diminish the phosphorylation levels of TOR under normal conditions. (A) The levels of phosphorylated TOR were measured by Western blot. The blot shown here is typical of three independent experiments. Rap, rapamycin (0.1 μM). (B) Quantification p-TOR from Western blot (A). These results are mean ± SD of three independent experiments. **P< 0.01. *p*-Values throughout were calculated (one-way ANOVA followed by a Student-Newman-Keuls test). Underlying data are available in S2 Table.
(TIF)

S11 Fig The mRNA levels of *ftn-1* and *ftn-2* are not altered after TOR is inhibited. Inactivation of TOR by *let-363* RNAi or rapamycin (Rap) treatment did not affect the mRNA levels of *ftn-1* and *ftn-2* in worms infected with *S*. Typhimurium (St) in the presence of ferric ammonium citrate (FAC, 100 μM). Con, control. These results are mean ± SD of five independent experiments. ns, not significant (one-way ANOVA followed by a Student-Newman-Keuls test). Underlying data are available in S2 Table.
(TIF)

S1 Table Sample sizes, median survival, the number of animals per experiment, and *P*-values for the *C. elegans* survival assays. (XLSX)

S2 Table Sample sizes, the number of animals per experiment, and *P*-values for the *C*. *elegans* pathogenesis and protein expression assays. (XLSX)
