# Supplementary figures and images for: TOR functions as a molecular switch connecting an iron cue with host innate defense against bacterial infection
Source: PLoS Genet. 2021 Mar 3;17(3):e1009383. doi: 10.1371/journal.pgen.1009383 (PMC7928448; doi:10.1371/journal.pgen.1009383)

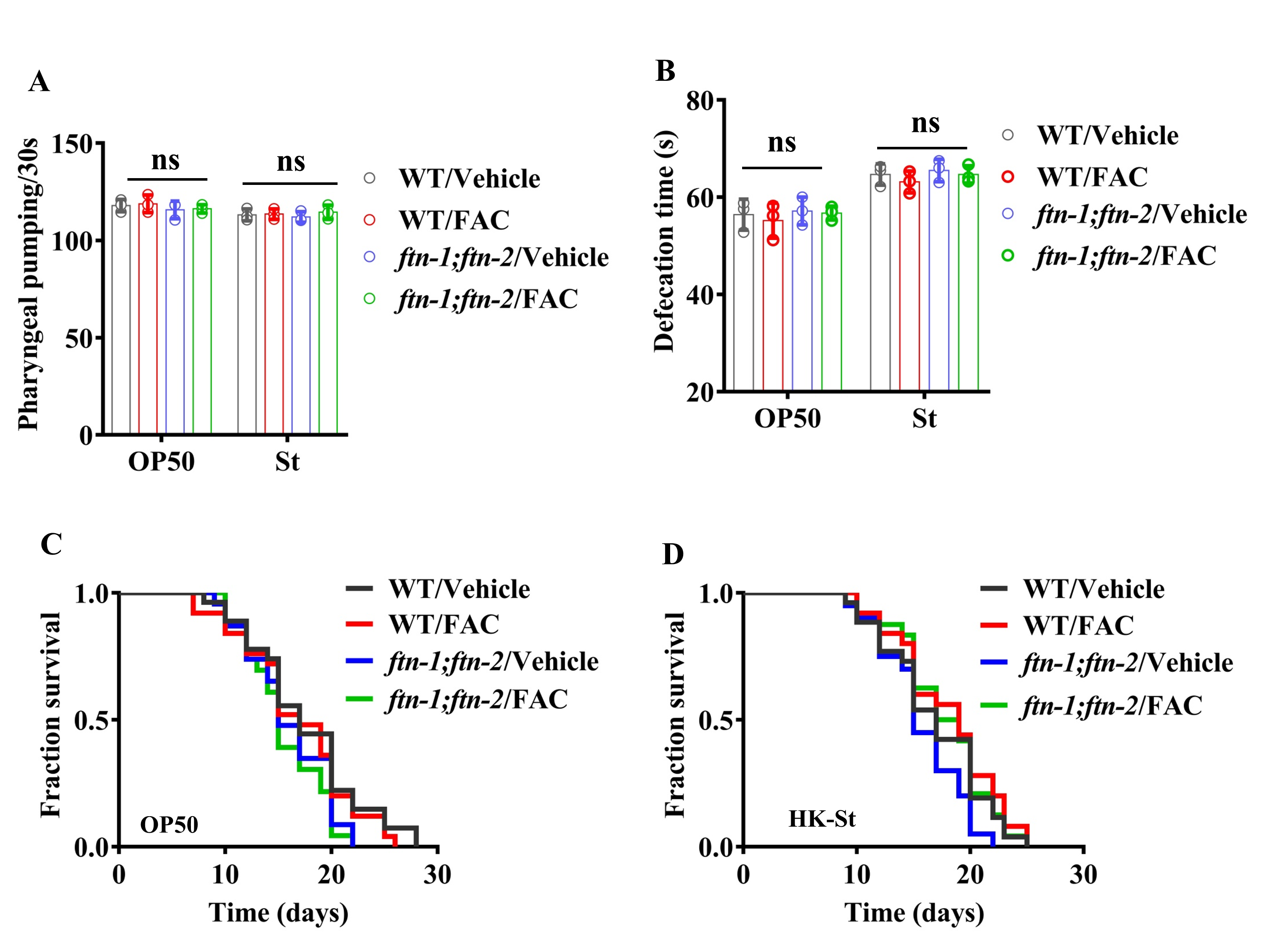

Supplement: S1 Fig — (A and B) FAC did not affect pharyngeal pumping and defecation rates in either WT worms or ftn-1(ok3625);ftn-2(ok404) double mutants grown on fed E. coli OP50 or exposed to S. Typhimurium (St). These results are mean ± SD of three independent experiments. ns, not significant (one-way ANOVA followed by a Student-Newman-Keuls test). Underlying data are available in S2 Table. (C) Ferric ammonium citrate (FAC, 100 μM) did not affect lifespan in either WT worms or ftn-1(ok3625);ftn-2(ok404) double mutants grown on fed E. coli OP50 (Log-rank test). Underlying data are available in S1 Table. (D) Lifespan of ftn-1(ok3625);ftn-2(ok404) double mutants grown on heat-killed S. Typhimurium (HK-St) in the presence of FAC was comparable to that of these mutants under normal conditions (Log-rank test). Underlying data are available in S1 Table. (TIF) [file pgen.1009383.s001.TIF]

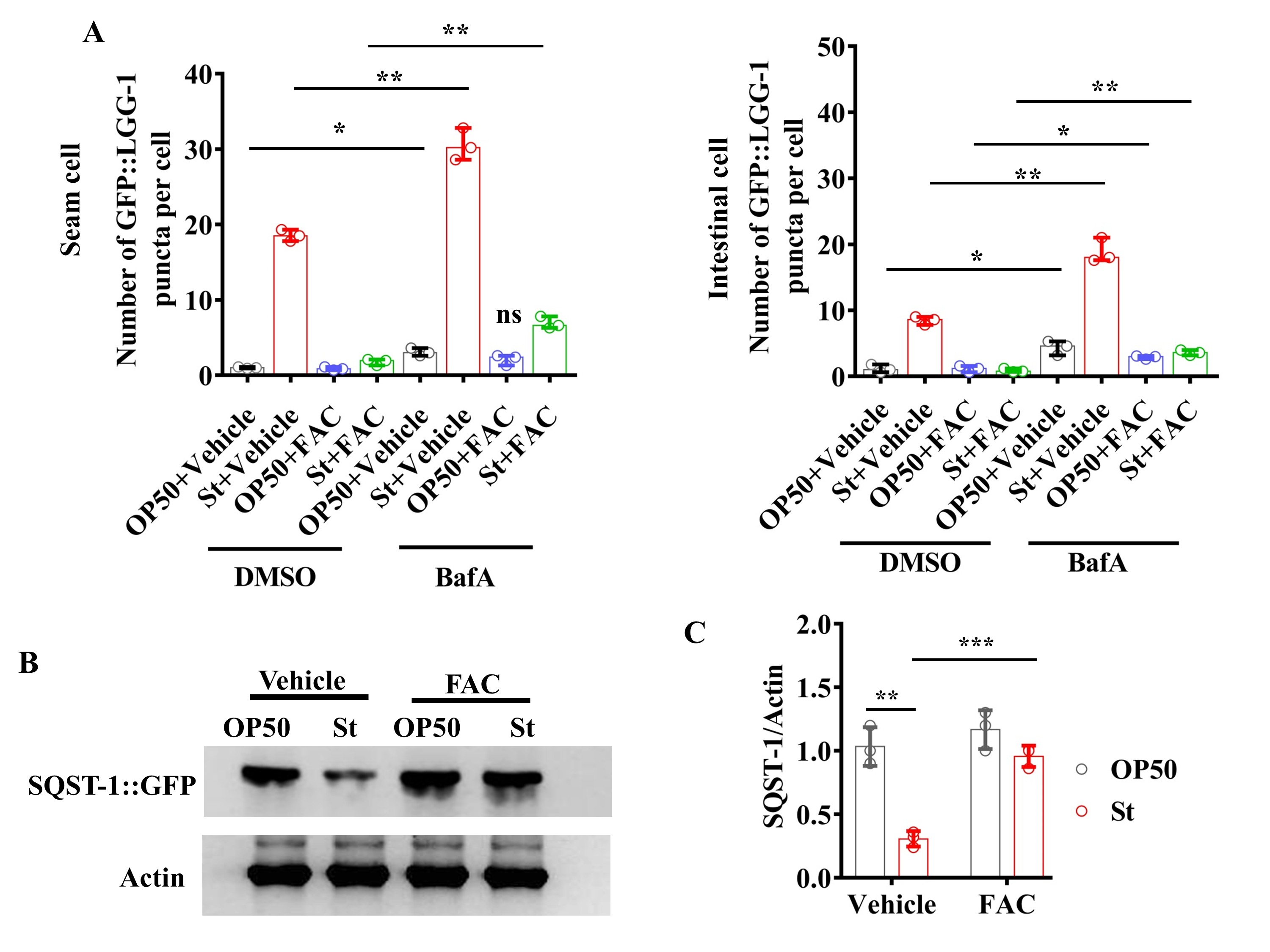

Supplement: S2 Fig — (A) The numbers of GFP::LGG-1 puncta were counted in the seam cells (left panel) and intestinal cells (right panel) of worms infected by S. Typhimurium in the absence or presence of ferric ammonium citrate (100 μM). FAC. After infected with S. Typhimurium or treated with FAC, the transgenic worms expressing GFP::LGG-1 were injected with 50 mM BafA or DMSO. These results are mean ± SD of three independent experiments (n = 15 worms per experiment). *P< 0.05; **P< 0.01. Underlying data are available in S2 Table. (B) The levels of SQST-1::GFP were measured by Western blot. The blot shown here is typical of three independent experiments. (C) Quantification SQST-1::GFP from Western blot (B). These results are mean ± SD of three independent experiments. **P< 0.01; ***P< 0.001. p-Values throughout were calculated using a one-way ANOVA followed by a Student-Newman-Keuls test. Underlying data are available in S2 Table. (TIF) [file pgen.1009383.s002.TIF]

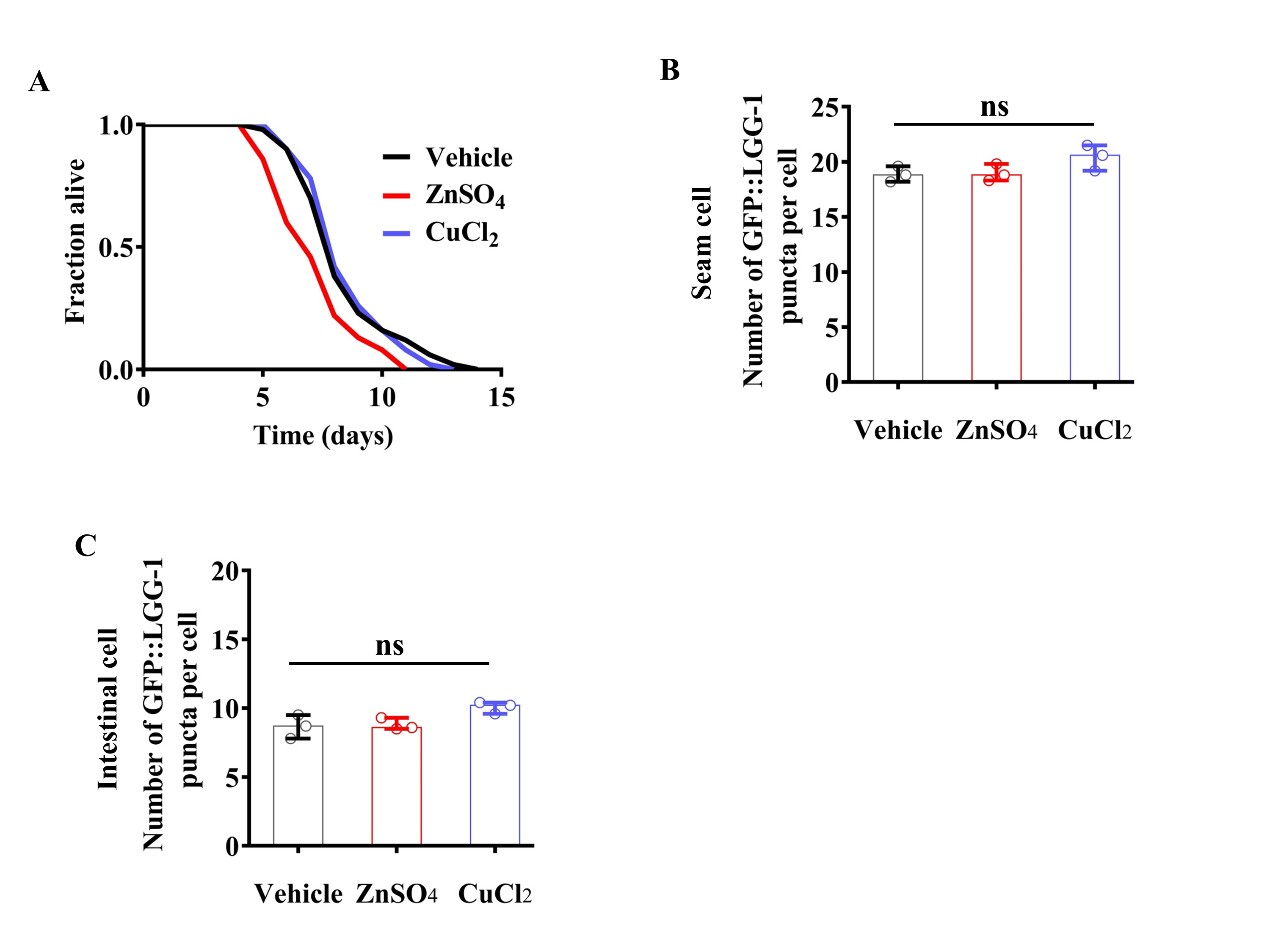

Supplement: S3 Fig — (A) Supplementation with CuCl2 (100 μM) did not influence survival rate (P> 0.05 vs vehicle), whereas supplementation with ZnSO4 (100 μM) slightly but significantly enhanced sensitivity of worms after S. Typhimurium infection (P< 0.05, ZnSO4 vs vehicle). p-Values throughout were calculated using a Log-rank test. Underlying data are available in S1 Table. (B and C) The numbers of GFP::LGG-1 puncta were counted in the seam cells (B) and intestinal cells (C) of worms exposed to S. Typhimurium for 12 h. These results are mean ± SD of three independent experiments (n = 15 worms per experiment). ns, not significant (one-way ANOVA followed by a Student-Newman-Keuls test). Underlying data are available in S2 Table. (TIF) [file pgen.1009383.s003.TIF]

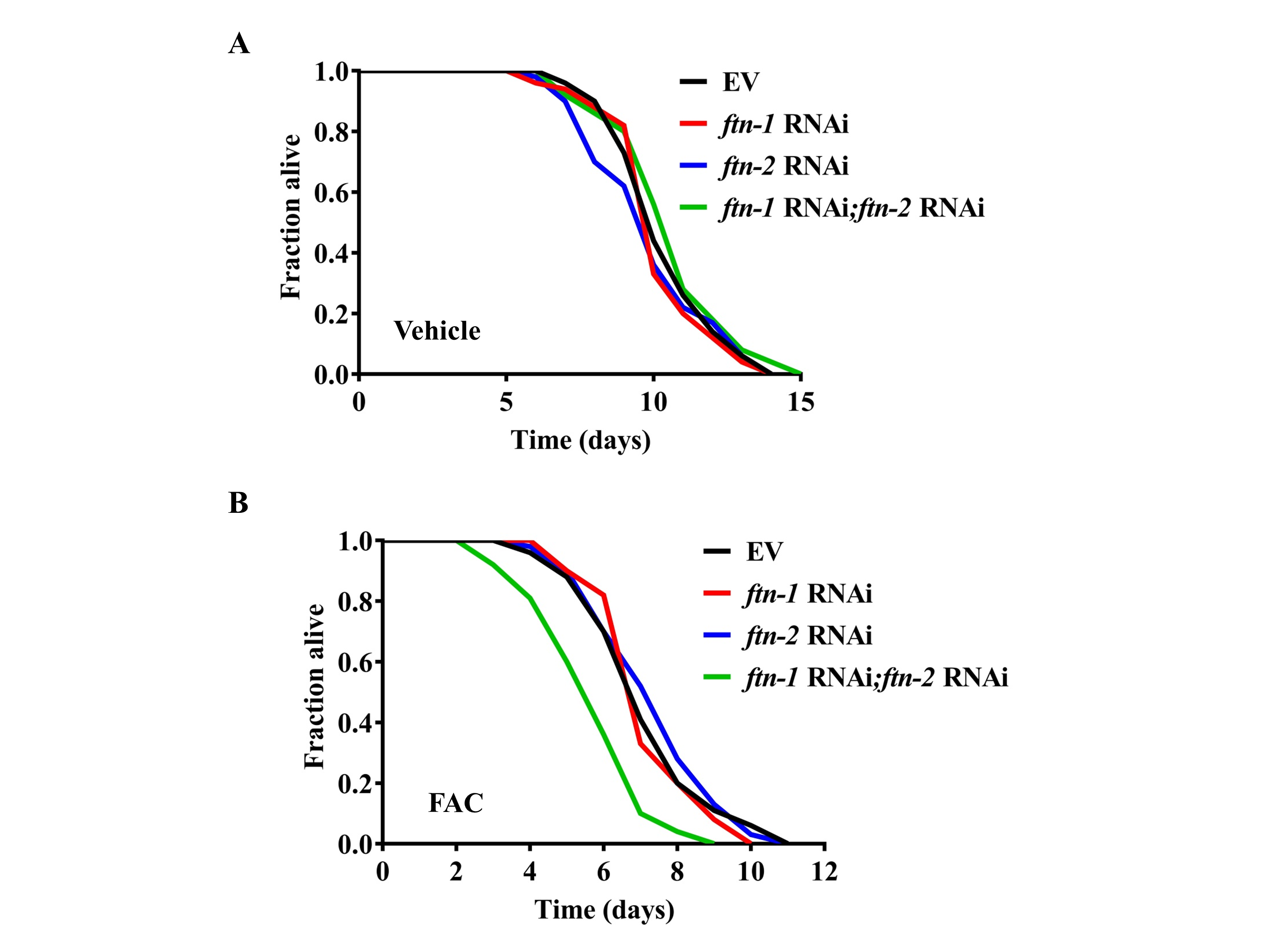

Supplement: S4 Fig — (A and B) The survival rates of worms subjected to ftn-1 RNAi, ftn-2 RNAi, or ftn-1;ftn-2 RNAi in the absence (A) or presence (B) of 100 μM ferric ammonium citrate (FAC, 100 μM) after S. Typhimurium infection. P< 0.01, ftn-1;ftn-2 RNAi relative to empty vector (EV) (B). p-Values throughout were calculated using a Log-rank test. Underlying data are available in S1 Table. (TIF) [file pgen.1009383.s004.TIF]

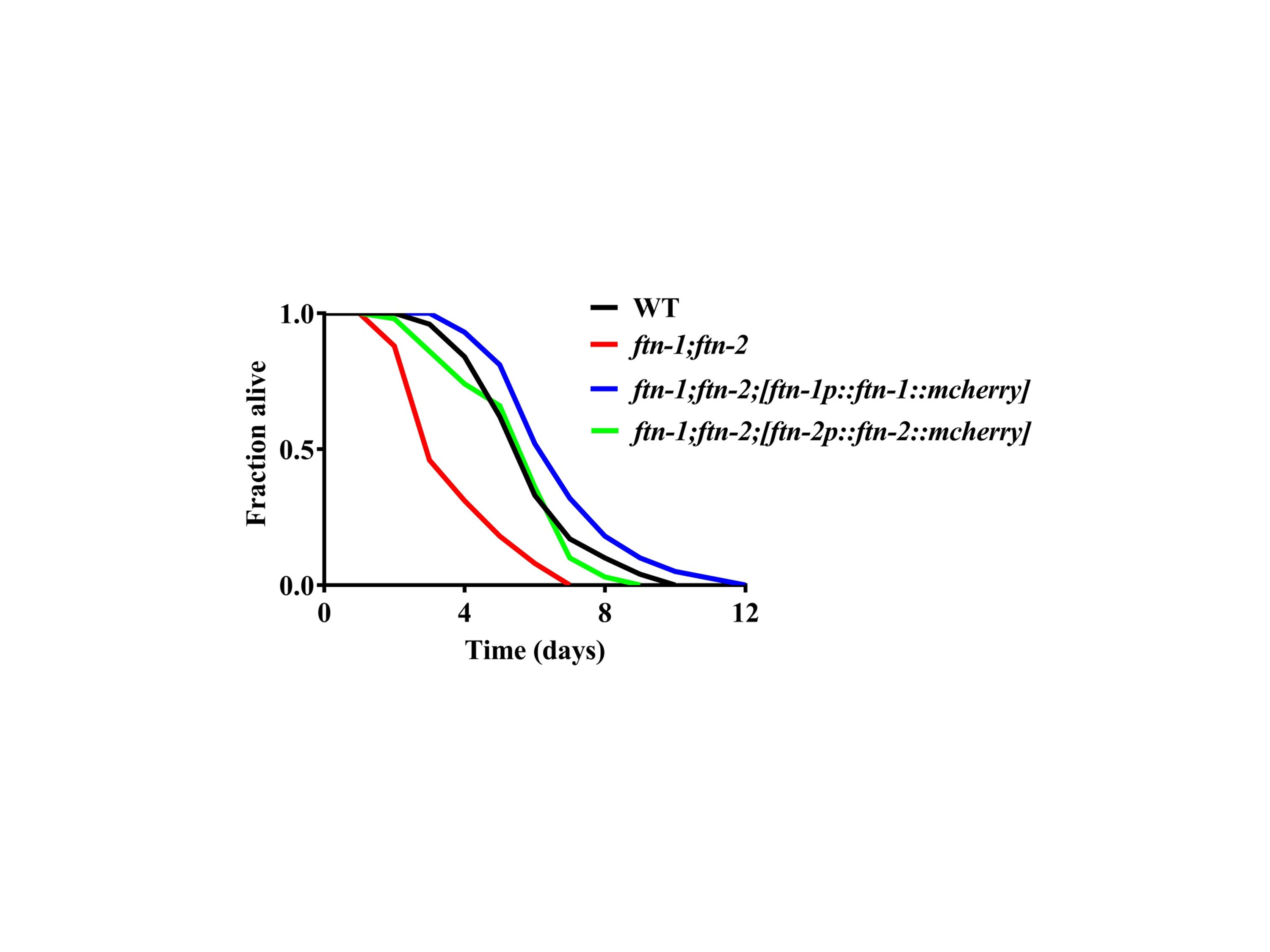

Supplement: S5 Fig — The concentration of ferric ammonium citrate (FAC) was 100 μM. P< 0.01 relative to ftn-1;ftn-2 (Log-rank test). Underlying data are available in S1 Table. (TIF) [file pgen.1009383.s005.TIF]

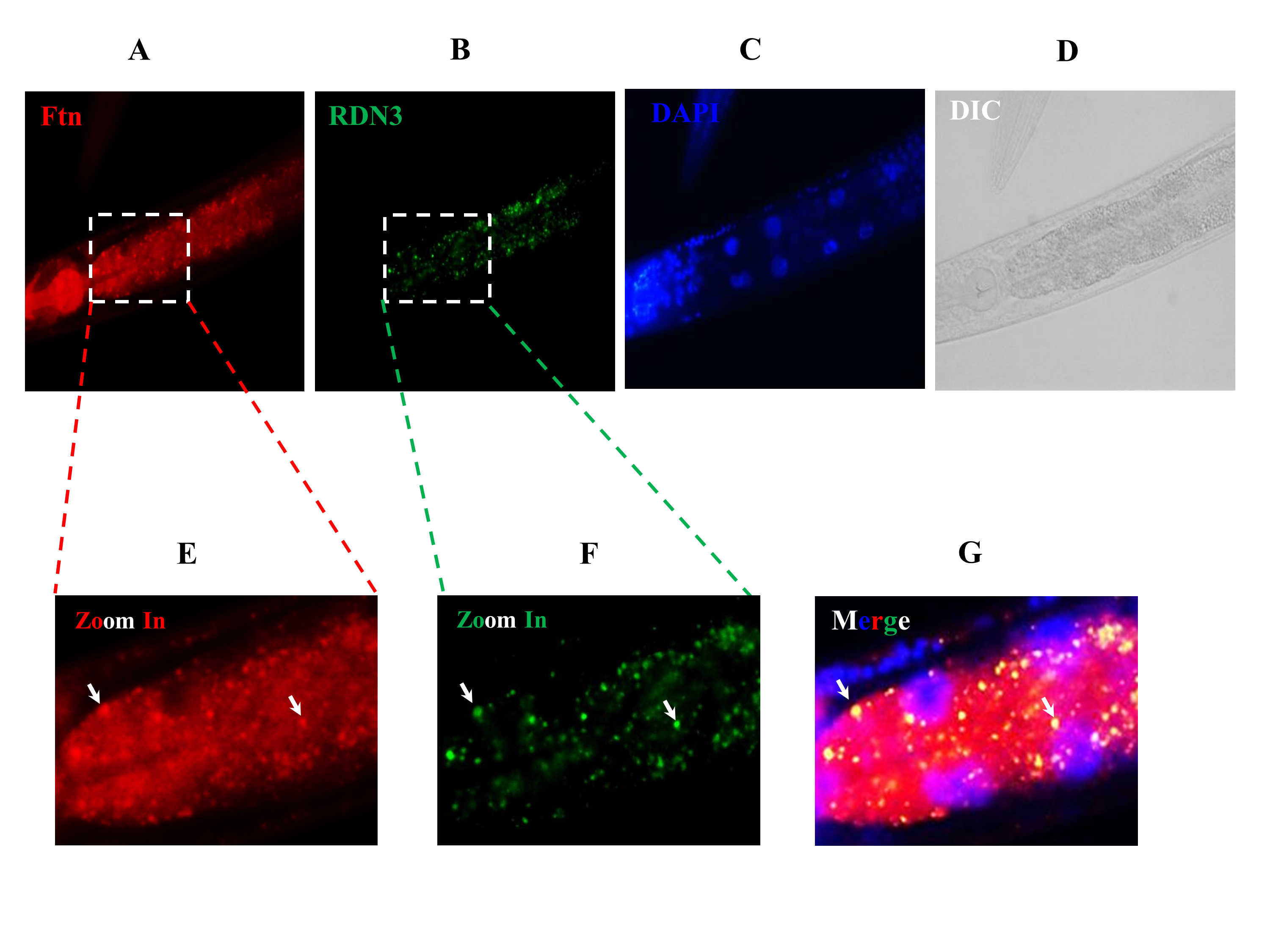

Supplement: S6 Fig — The ferric foci stained by RDN3, a ferric (Fe3+)-selective fluorescent sensor, were merged into ferritin detected by immunofluorescence. (A) Immunofluorescence with anti-Ftn antibodies; (B) RDN3 staining; (C) DAPI staining; (D) DIC image; (E and F) Enlarged views were shown for areas. (G) Merged image. The arrows denote representative ferric foci. (TIF) [file pgen.1009383.s006.TIF]

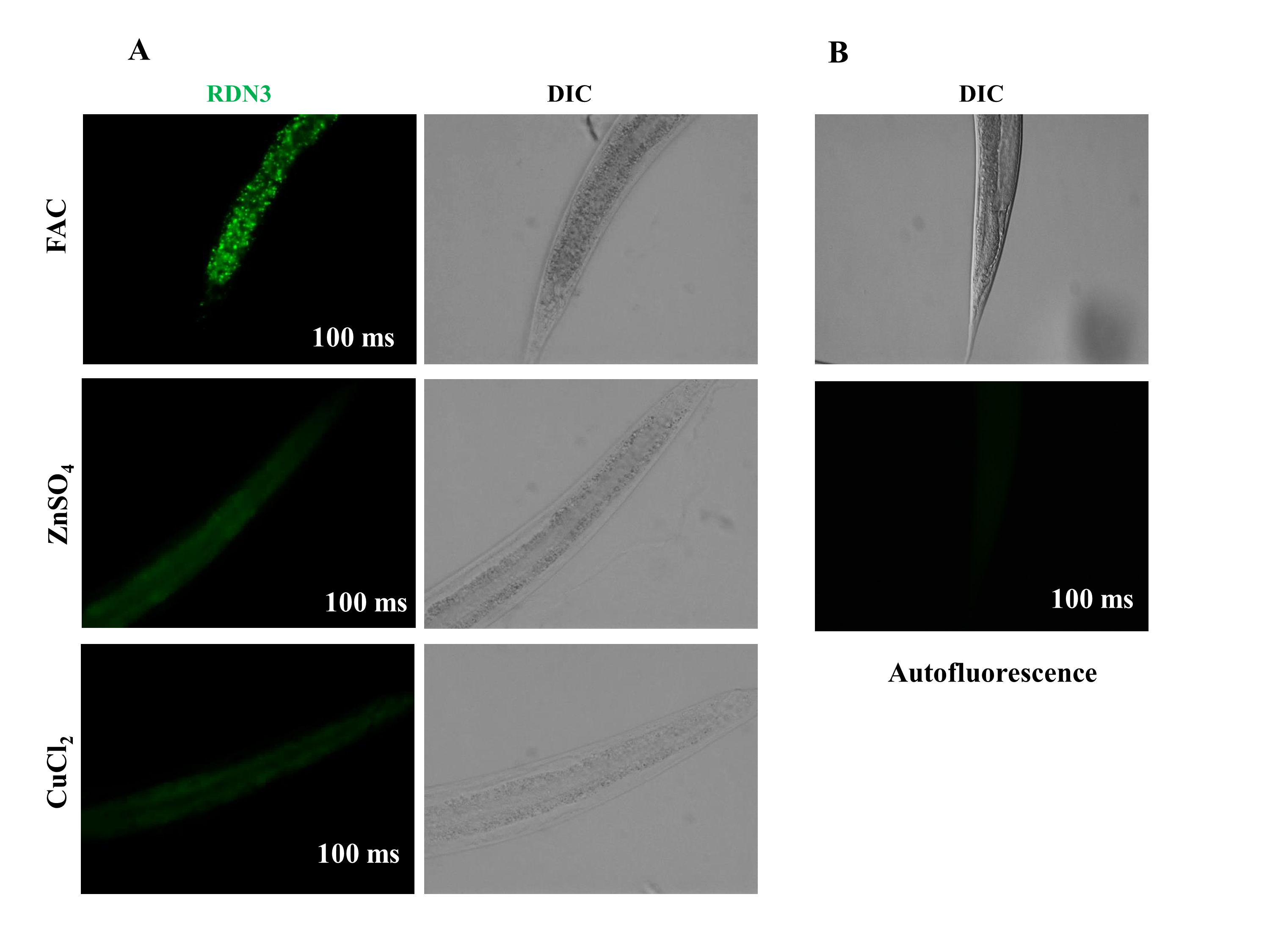

Supplement: S7 Fig — (A) Worms were stained with RDN3 in the presence of Zn2+ and Cu2+. (B) Autofluorescence in worms without RDN3 under normal conditions. (TIF) [file pgen.1009383.s007.TIF]

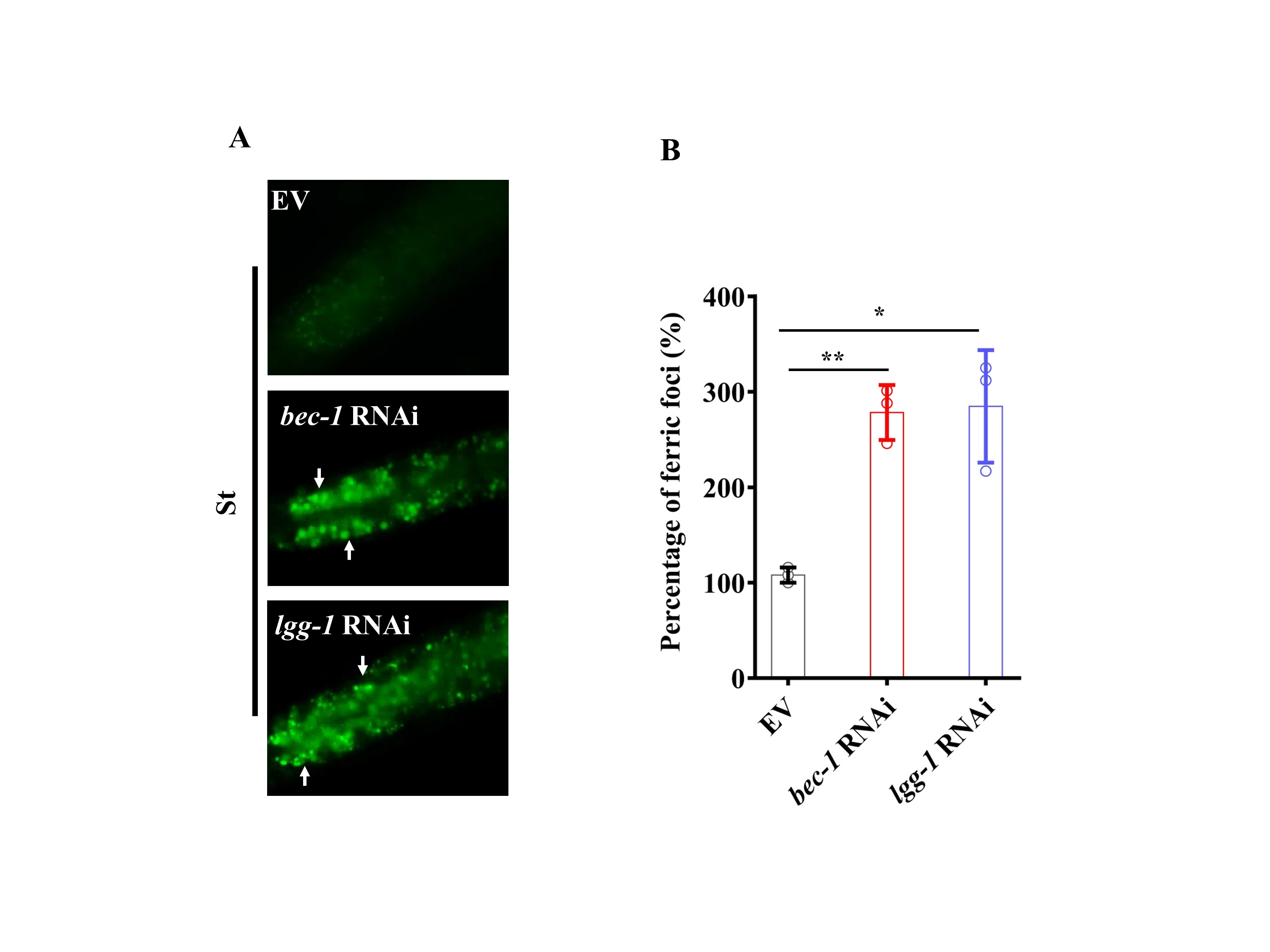

Supplement: S8 Fig — (A) Worms were stained by RDN3 staining upon S. Typhimurium infection. The arrows denote representative ferric foci. (B) Percentage of ferric foci. These results are mean ± SD of three independent experiments. *P< 0.05; **P< 0.01 relative to empty vector (EV) (one-way ANOVA followed by a Student-Newman-Keuls test). Underlying data are available in S2 Table. (TIF) [file pgen.1009383.s008.TIF]

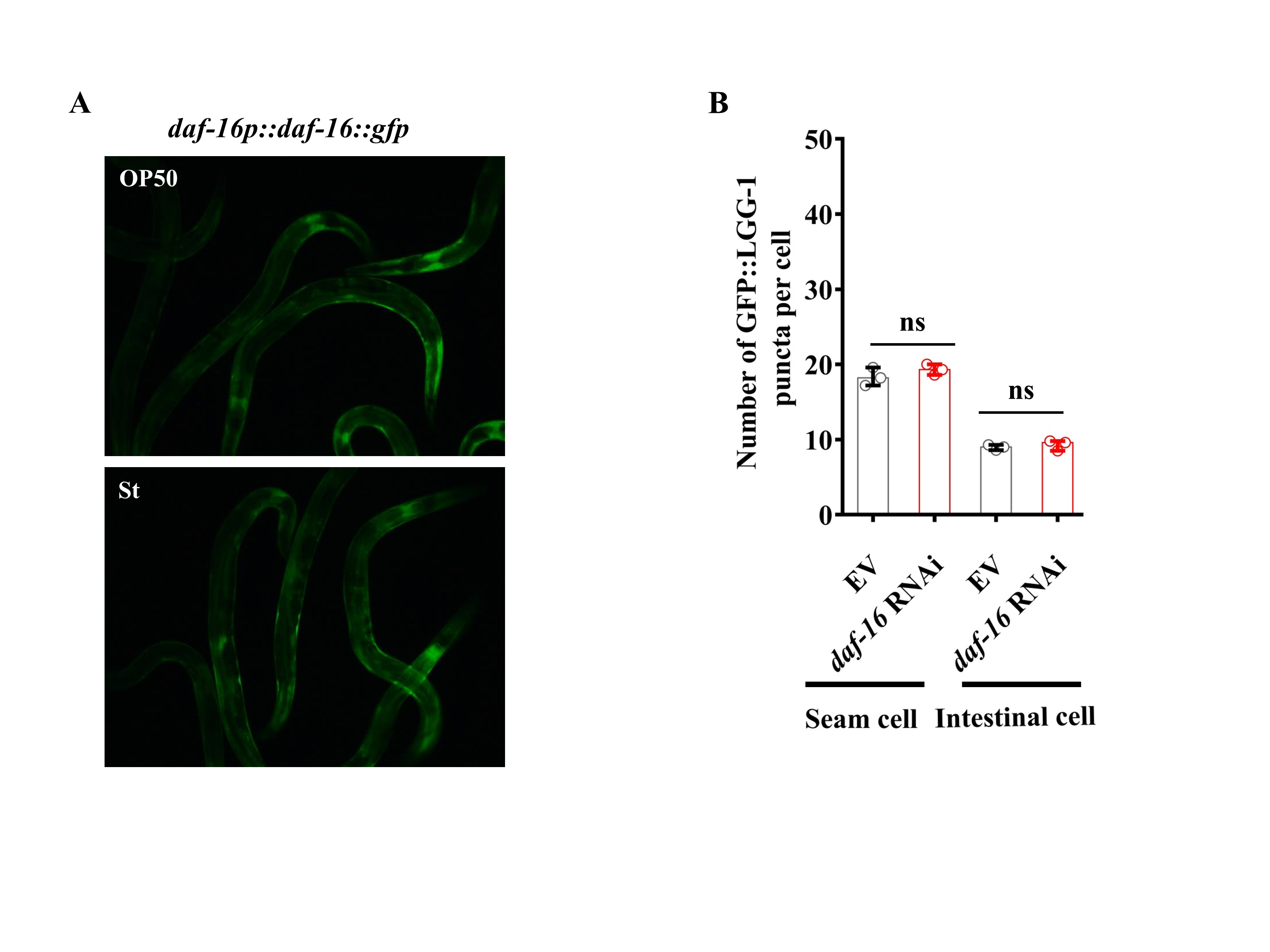

Supplement: S9 Fig — (A) S. Typhimurium (St) infection did not induce nuclear translocation of DAF-16. (B) Knockdown of daf-16 by RNAi did not influence autophagy in worms after S. Typhimurium infection. The numbers of GFP::LGG-1 puncta were counted in the seam cells and intestinal cells of worms. These results are mean ± SD of three independent experiments (n = 15 worms per experiment). ns, not significant (two sample t-test). Underlying data are available in S2 Table. (TIF) [file pgen.1009383.s009.TIF]

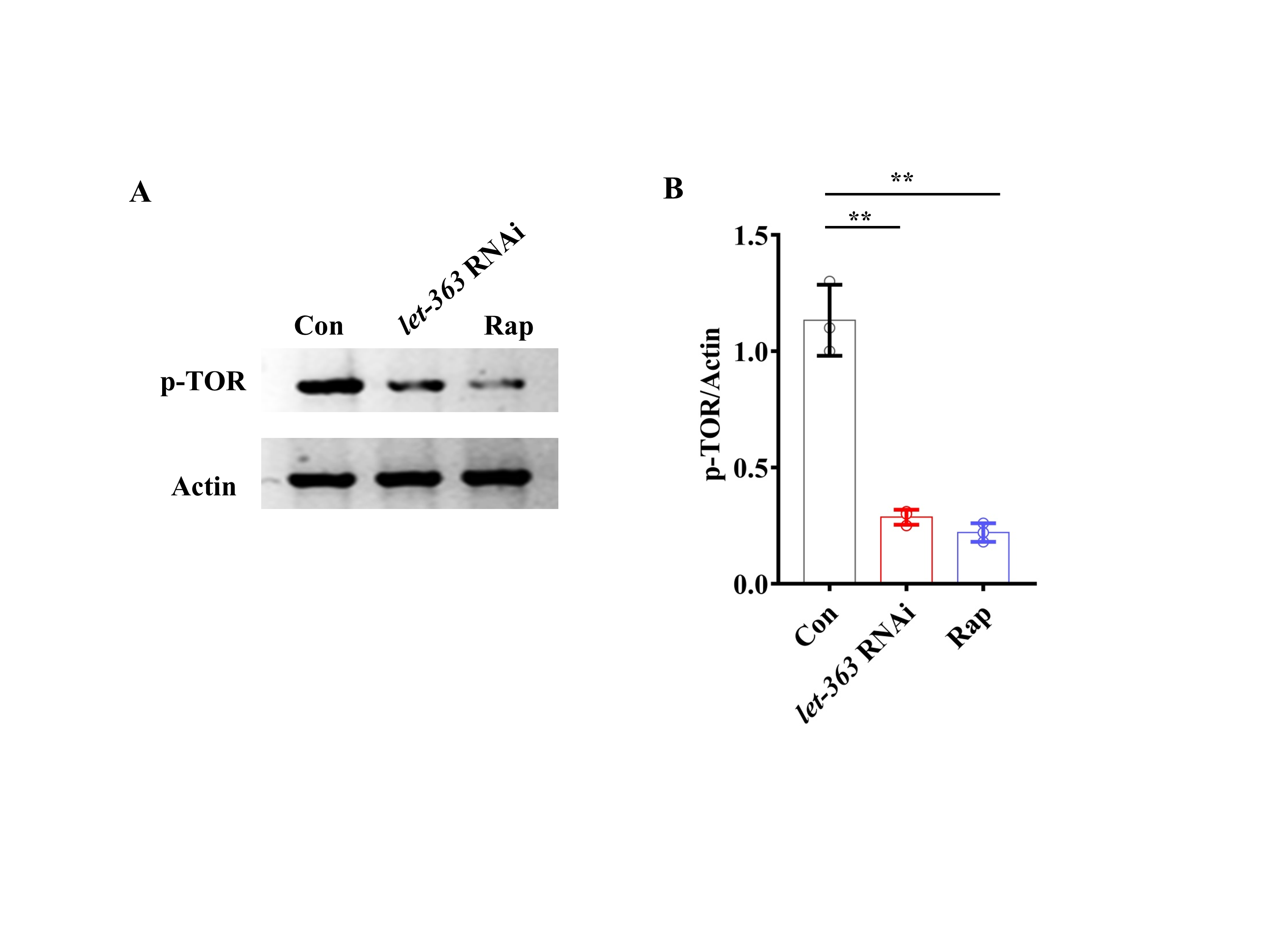

Supplement: S10 Fig — (A) The levels of phosphorylated TOR were measured by Western blot. The blot shown here is typical of three independent experiments. Rap, rapamycin (0.1 μM). (B) Quantification p-TOR from Western blot (A). These results are mean ± SD of three independent experiments. **P< 0.01. p-Values throughout were calculated (one-way ANOVA followed by a Student-Newman-Keuls test). Underlying data are available in S2 Table. (TIF) [file pgen.1009383.s010.TIF]

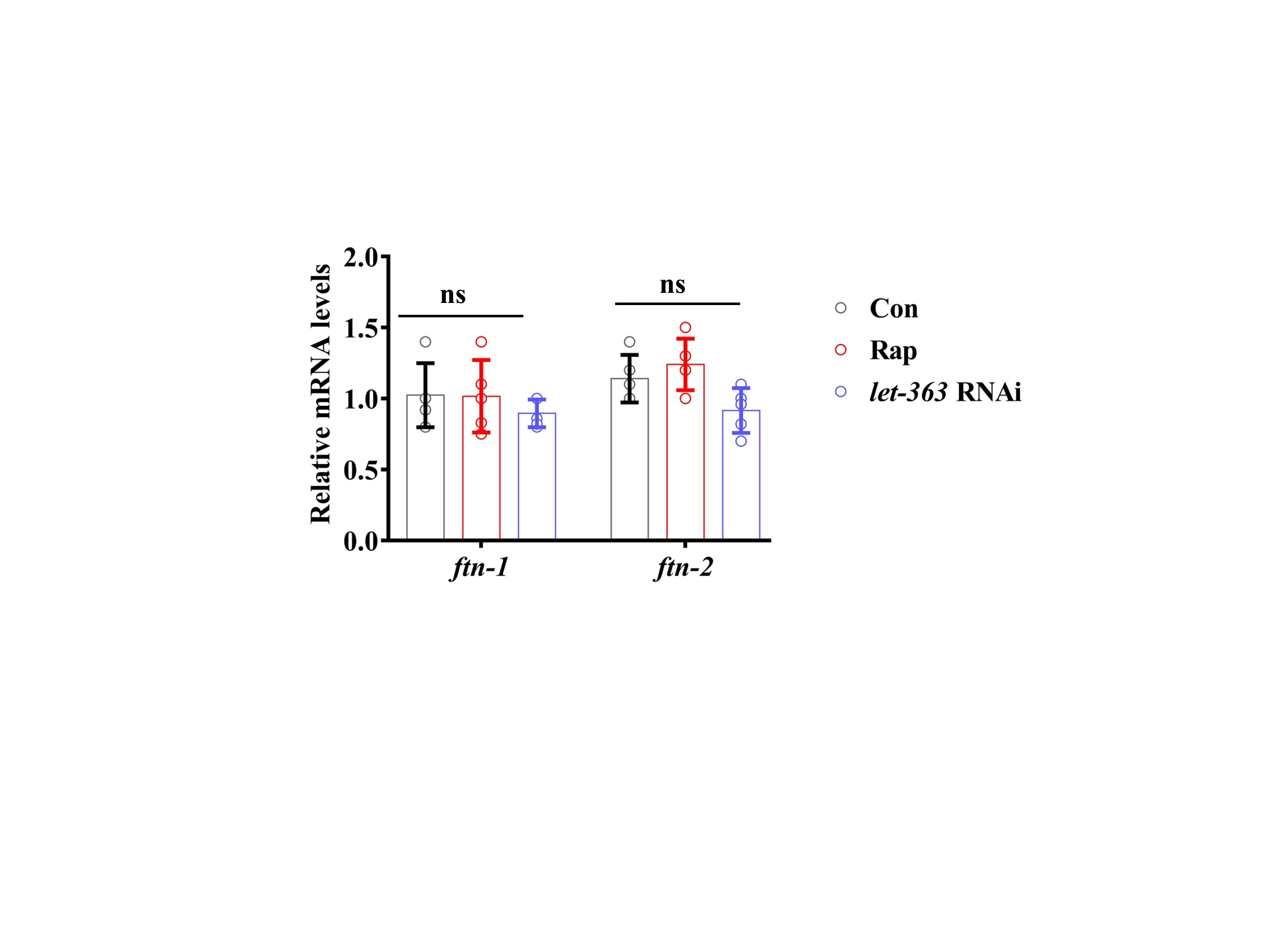

Supplement: S11 Fig — Inactivation of TOR by let-363 RNAi or rapamycin (Rap) treatment did not affect the mRNA levels of ftn-1 and ftn-2 in worms infected with S. Typhimurium (St) in the presence of ferric ammonium citrate (FAC, 100 μM). Con, control. These results are mean ± SD of five independent experiments. ns, not significant (one-way ANOVA followed by a Student-Newman-Keuls test). Underlying data are available in S2 Table. (TIF) [file pgen.1009383.s011.TIF]
